# Preventive and therapeutic benefits of nelfinavir in rhesus macaques and human beings infected with SARS-CoV-2

**DOI:** 10.1038/s41392-023-01429-0

**Published:** 2023-04-24

**Authors:** Zhijian Xu, Danrong Shi, Jian-Bao Han, Yun Ling, Xiangrui Jiang, Xiangyun Lu, Chuan Li, Likun Gong, Guangbo Ge, Yani Zhang, Yi Zang, Tian-Zhang Song, Xiao-Li Feng, Ren-Rong Tian, Jia Ji, Miaojin Zhu, Nanping Wu, Chunhui Wu, Zhen Wang, Yechun Xu, Cheng Peng, Min Zheng, Junling Yang, Feifei Du, Junliang Wu, Peipei Wang, Jingshan Shen, Jianliang Zhang, Yong-Tang Zheng, Hangping Yao, Weiliang Zhu

**Affiliations:** 1 State Key Laboratory of Drug Research, Shanghai Institute of Materia Medica, Chinese Academy of Sciences, Shanghai, 201203 China; 2 Drug Discovery and Design Center, Shanghai Institute of Materia Medica, Chinese Academy of Sciences, Shanghai, 201203 China; 3 School of Pharmacy, University of Chinese Academy of Sciences, Beijing, 100049 China; 4 State Key Laboratory for Diagnosis and Treatment of Infectious Diseases, National Clinical Research Center for Infectious Diseases, First Affiliated Hospital, Zhejiang University School of Medicine, Hangzhou, 310003 China; 5 Kunming National High-level Biosafety Research Center for Non-human Primates, Center for Biosafety Mega-Science, Kunming Institute of Zoology, Chinese Academy of Sciences, Kunming, Yunnan 650107 China; 6 Department of Infectious Disease, Shanghai Public Health Clinical Center, Fudan University, Shanghai, 201508 China; 7 Center for Drug Safety Evaluation and Research, Shanghai Institute of Materia Medica, Chinese Academy of Sciences, Shanghai, 201203 China; 8 Shanghai Frontiers Science Center of TCM Chemical Biology, Institute of Interdisciplinary Integrative Medicine Research, Shanghai University of Traditional Chinese Medicine, Shanghai, 201203 China; 9 Key Laboratory of Animal Models and Human Disease Mechanisms of the Chinese Academy of Sciences, KIZ-CUHK Joint Laboratory of Bioresources and Molecular Research in Common Diseases, Kunming Institute of Zoology, Chinese Academy of Sciences, Kunming, Yunnan 650223 China; 10 Jinan Microecological Biomedicine Shandong Laboratory, Jinan, 250117 China

**Keywords:** Molecular medicine, Translational research

## Abstract

Effective drugs with broad spectrum safety profile to all people are highly expected to combat COVID-19 caused by SARS-CoV-2. Here we report that nelfinavir, an FDA approved drug for the treatment of HIV infection, is effective against SARS-CoV-2 and COVID-19. Preincubation of nelfinavir could inhibit the activity of the main protease of the SARS-CoV-2 (IC_50_ = 8.26 μM), while its antiviral activity in Vero E6 cells against a clinical isolate of SARS-CoV-2 was determined to be 2.93 μM (EC_50_). In comparison with vehicle-treated animals, rhesus macaque prophylactically treated with nelfinavir had significantly lower temperature and significantly reduced virus loads in the nasal and anal swabs of the animals. At necropsy, nelfinavir-treated animals had a significant reduction of the viral replication in the lungs by nearly three orders of magnitude. A prospective clinic study with 37 enrolled treatment-naive patients at Shanghai Public Health Clinical Center, which were randomized (1:1) to nelfinavir and control groups, showed that the nelfinavir treatment could shorten the duration of viral shedding by 5.5 days (9.0 vs. 14.5 days, *P* = 0.055) and the duration of fever time by 3.8 days (2.8 vs. 6.6 days, *P* = 0.014) in mild/moderate COVID-19 patients. The antiviral efficiency and clinical benefits in rhesus macaque model and in COVID-19 patients, together with its well-established good safety profile in almost all ages and during pregnancy, indicated that nelfinavir is a highly promising medication with the potential of preventative effect for the treatment of COVID-19.

## Introduction

Severe acute respiratory syndrome coronavirus 2 (SARS-CoV-2), a highly infectious positive-strand RNA virus causing COVID-19 (coronavirus disease 2019), has evolved through Alpha, Beta, Gamma, Delta, and Omicron mutant strains, resulting in an unabated global pandemic trend. Although quite a few COVID-19 vaccines have been used worldwide since 2021, which could significantly protect people from getting severely ill or dying, as many as hundreds of thousands of infection cases are currently reported each day.^1^ Therefore, effective drugs with good safety profiles are highly expected to protect the SARS-CoV-2 infected people by stopping the virus replication in the body to prevent the people from becoming severe or dying. There are two well known drug targets for SARS-CoV-2, one is RNA-dependent RNA polymerase (RdRp) and the other is main protease (M^pro^). Replication of RNA from an RNA template is catalyzed by RdRp, leading to its vital role in SARS-CoV-2 replication and transcription. M^pro^, also known as 3C-like protease (chymotrypsin-like protease), cleaving at least 11 sites of SARS-CoV-2 polyprotein, is highly conservable among coronaviruses. Sequence alignment revealed that the M^pro^ of SARS-CoV-2 shares 96% similarity with that of SARS-CoV-1. In addition, M^pro^ has no human homolog, making M^pro^ the most attractive drug target to treat COVID-19.

Small molecule antivirals have the advantages of easy manufacture, low cost and good accessibility.^2–4^ There are several small molecule drugs shown beneficial effects in preclinical and clinical trials. For example, in the Evaluation of Protease Inhibition for Covid-19 in High-Risk Patients (EPIC-HR) double-blind controlled trial with a total of 2246 unvaccinated patients from July 16 to December 9, 2021 when the Delta mutant dominated, nirmatrelvir, an orally M^pro^ inhibitor,^5^ was found to be able to decrease the probability of hospitalization or mortality by 89% in adults at high risk who were not hospitalized.^6^ Remdesivir, an intravenously administered RdRp inhibitor, could reduce recovery time by 5 days in hospitalized adults.^7^ Although remdesivir could reduce virus titers in bronchoalveolar lavages in a SARS-CoV-2 infected rhesus macaque model, it didn’t reduce virus shedding.^8^ VV116, an oral RdRp inhibitor with favorable features for safety, tolerability, and pharmacokinetics, has been approved in Uzbekistan.^9^ In a double-blind randomized trial (MOVe-OUT) with 1433 participants between May 6, 2021 and November 4, 2021 when the delta mutant dominated, molnupiravir, another oral RdRp inhibitor, decreased the risk of hospitalization or death by 48% in patients who were not hospitalized.^10^ Dexamethasone, a corticosteroid medication not directly acting on SARS-CoV-2, could lower 28-day mortality in severe patients but not among those receiving no respiratory support.^11^

However, currently available drugs do not mean we can beat the virus efficiently. For example, nirmatrelvir must be co-packaged with ritonavir, an inhibitor of cytochrome P450 (CYP) 3A4, which would result in drug-drug interactions with many commonly used medications. In the real world for patients aged 40 to 64 years old, a retrospective observational cohort study for nirmatrelvir between January 9, 2022 and March 31, 2022 with the omicron mutant as the most prevalent in Israel, the rate of hospitalization owing to COVID-19 was 15.2 instances per 100,000 person-days among treated patients and 15.8 cases per 100,000 person-days among untreated patients, demonstrating nirmatrelvir is not effective against SARS-CoV-2 in younger adults.^12^ Molnupiravir might cause mutations in human DNA,^13^ which prevent its use during pregnancy. The largest randomized trial so far for molnupiravir in the real world, Platform Adaptive trial of NOvel antiviRals for eArly treatMent of COVID-19 in the Community (PANORAMIC) with 12,529 patients in the molnupiravir plus usual care group and 12,525 patients in the control group, showed that hospitalizations and deaths from COVID-19 were not reduced by molnupiravir in vaccinated high-risk individuals.^14^ Cytokine storm, also called hypercytokinemia, is an extreme immunological response characterized by the rapid release of too many cytokines into the blood. The COVID-19 illness severity has been linked to the cytokine storm, which could lead to acute respiratory distress syndrome.^15^ The limited success of nirmatrelvir and molnupiravir in the real world could be partly attributed to their ability to suppress only SARS-CoV-2 activity but not the accompanying cytokine storm. In addition, COVID-19 vaccine is not recommended to anyone who has ever had a life-threatening reaction to any of the components of the COVID-19 vaccine. In this particular scenario, a medication that has a preventative effect is required. Therefore, effective drugs with well proved safety profile to people of all ages and during pregnancy, are urgently needed to combat COVID-19.

Herein, inspired by cutting-edge computer-aided design (CADD) techniques as well as expert knowledge, following enzymatic inhibition assay, cellular activity assay, animal investigation and clinical trial, we report the discovery of nelfinavir, an FDA approved antiretroviral drug, as a potential medication for COVID-19 with potential of preventative effect for almost all ages of people. Monkeys given nelfinavir prophylactically showed significantly lower temperatures and virus loads in nasal and anal swabs, as well as a significant reduction in virus replication in the lungs by nearly three orders of magnitude. In patients with mild/moderate COVID-19, nelfinavir could reduce the length of viral shedding by 5.5 days and the length of fever period by 3.8 days.

## Results

### Systemic exposure of healthy rhesus macaques to nelfinavir

Combining in silico (Supplementary Tables S1-5) and in vitro studies, nelfinavir, an FDA approved drug for the treatment of HIV infection, is effective against SARS-CoV-2 and COVID-19 (Supplementary Fig. S1-S3). Preincubation of nelfinavir could inhibit the SARS-CoV-2 main protease activity (IC_50_ = 8.26 μM), while its antiviral activity in Vero E6 cells against a clinical isolate of SARS-CoV-2 was determined to be 2.93 μM (EC_50_, Table 1).

**Table 1:** The EC_50_ (μM, mean ± sd) of the drugs against different SARS-CoV-2 mutants

Drugs	Cell Lines	Wild Type	Delta	Omicron
Nelfinavir	Vero E6	2.93 ± 0.32	2.09 ± 0.23	2.03 ± 0.12
Nelfinavir	Calu-3	1.86 ± 0.26	1.76 ± 0.21	1.73 ± 0.08
Remdesivir	Vero E6	1.01 ± 0.13	0.85 ± 0.10	1.50 ± 0.20
Remdesivir	Calu-3	1.11 ± 0.17	1.11 ± 0.12	1.50 ± 0.07
Paxlovid	Vero E6	0.85 ± 0.16	1.22 ± 0.09	1.84 ± 0.05
Paxlovid	Calu-3	1.62 ± 0.2	0.73 ± 0.08	2.48 ± 0.26

**Fig. 1:**
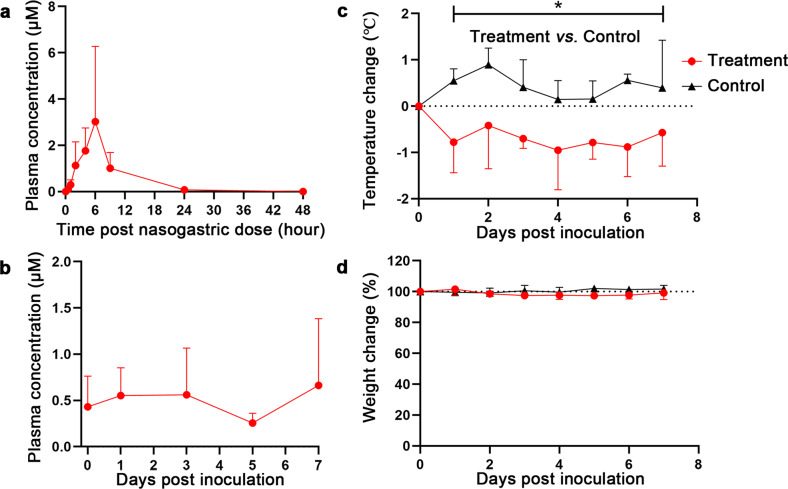
Nelfinavir plasma exposure and clinical benefits in rhesus macaques inoculated with SARS-CoV-2. **a** Plasma concentrations of nelfinavir in two healthy rhesus macaques plotted versus time (hours). **b** Concentration of nelfinavir measured in blood plasma in three rhesus macaques infected with SARS-CoV-2 strain 20SF107. Blood samples were collected 5 h after nelfinavir administration at 0, 1, 3, and 7 dpi, and before nelfinavir administration at 5 dpi, respectively. **c**, **d** Temperature (**c**) and body weight (**d**) changes in SARS-CoV-2 infected rhesus macaques treated with nelfinavir. SARS-CoV-2 strain 20SF107 was inoculated into two groups of three rhesus macaques, among which one group was nasogastric administered 5 mL/kg vehicle solution (0.5% CMC-Na; vehicle control; black triangles) and the other group was administered 200 mg/kg nelfinavir starting 1 day before inoculation (prophylactic nelfinavir; red circles). After inoculation, the animals were observed once daily for temperature and body weight changes. Asterisks indicate statistically significant difference in a mixed ANOVA between the vehicle control and prophylactic nelfinavir groups. *P < 0.05

Two healthy rhesus macaques received nelfinavir at 200 mg/kg via nasogastric administration route. The average maximum plasma concentration (*C*_max_) of nelfinavir was 3.20 μM, which achieved at 5 h after dosing, with a large inter-animal difference (Supplementary Table S6 and Fig. 1a). The *C*_max_ in rhesus macaques was comparable to the EC_50_ of nelfinavir against the virus in Vero E6 cells (2.93 μM). Considering the higher plasma exposure of nelfinavir in human (refer to Discussion for details), the dose of nelfinavir 200 mg/kg was used in rhesus macaques infected with SARS-CoV-2.

### Systemic exposure of SARS-CoV-2-infected rhesus macaques to nelfinavir

A group of 3 rhesus macaques received nelfinavir (suspended in 0.5%CMC-Na) at 200 mg/kg via nasogastric administration route. After inoculation, the nelfinavir treatment was continued twice daily. The plasma concentrations of nelfinavir at 5 h after dosing (C_5h_) at -1, 0, 1, 3, and 7 dpi and immediately before the first dose at 5 dpi (C_trough_) were determined. In SARS-CoV-2 infected monkeys, C_5h_ of nelfinavir averaged 0.55 μM and trough plasma concentrations were 0.26 μM (Fig. 1b), which was only around 1/6 of the plasma concentrations in the healthy rhesus macaques.

### Significantly reduced temperature in the treated rhesus macaques

**Fig. 2:**
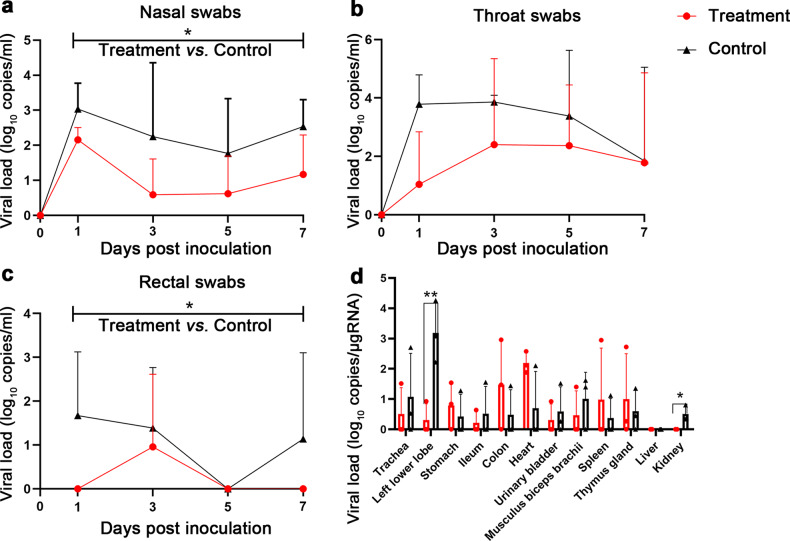
**a**, **b**, **c**, **d** Viral loads in nasal (**a**), throat (**b**), rectal (**c**) swabs, and tissues (**d**) collected from SARS-CoV-2-infected rhesus macaques treated with nelfinavir (*n* = 3) or vehicle solution (*n* = 3). A 2-way mixed ANOVA (**a**, **b**, and **c**) or unpaired *t*-test (**d**) was used for statistical analysis

With another group of 3 rhesus macaques as vehicle control (0.5%CMC-Na), the temperatures and body weights of the control and infected monkeys were monitored every day until euthanasia. Elevated body temperatures were detected in most days in the monkeys of the control group after inoculation (Fig. 1c). The temperatures in nelfinavir-treated monkeys were significantly lower than the control monkeys starting from 1 dpi (Fig. 2c). The body weight change is not significant between the nelfinavir-treated and the control monkeys (Fig. 1d).

### Significantly reduced virus loads in the treated rhesus macaques

Nasal, throat, and rectal swabs were collected at 0, 1, 3, 5, and 7 dpi. Significant reductions in viral load were observed in the nasal and rectal swabs collected from nelfinavir-treated monkeys compared to the control group (Fig. 2a, c). However, the reduction trend in viral load in throat swabs was observed without significance (Fig. 2b).

On 8 dpi, all monkeys were euthanized. Tissue samples were collected from nelfinavir-treated and vehicle-treated monkeys to compare virus replication. In kidney samples from nelfinavir-treated monkeys, the virus RNA could not be detected while that in the vehicle-treated monkeys is detectable (Fig. 2d). In lung (left lower lobe) samples from nelfinavir-treated monkeys, the virus RNA reduced 2.88 log compared to vehicle-treated monkeys (Fig. 2d). These data show that the viral load in the kidneys and lungs of the nelfinavir-treated animals was significantly lower than that in the vehicle-treated controls.

We found that the viral loads in several extrapulmonary tissues, e.g., colon and heart, in nelfinavir treatment group have increasing trends in comparison with the control group. However, no statistical difference was found between the two groups (Fig. 2d). Nevertheless, this phenomenon deserves further attention in future study.

### Brief information of the nelfinavir-treated and control groups in clinical trial

To assess the efficacy and safety of nelfinavir in treating patients with mild or moderate COVID-19 infection, we prospectively recruited treatment-naive COVID-19 patients at Shanghai Public Health Clinical Center. The ethical committee of Shanghai Public Health Clinical Center has authorized this study (YJ-2020-E043-02). Between 2020-07-14 and 2021-04-02, out of 44 patients screened, 38 met the criteria for eligibility. Nelfinavir was given to 19 patients, while basic treatment was given to 19 patients, according to randomization. One patient in the control group did not receive the basic treatment after randomization, so 19 and 18 patients were included in FAS/PPS/SS. The age, body weight, sex, ethnic group, disease severity (Supplementary Table S7), and the past medical history (Supplementary Table S8) were all comparable without statistic difference between the nelfinavir-treated and the control groups. In addition, the history of food and drug allergy, comorbidities and treatment history, physical examination, vital signs, SARS-CoV-2 RNA detection, SARS-CoV-2 IgG and IgM antibody detection, infectious disease serological examination, 12-lead electrocardiogram, TTCR assessment, chest CT examination, and liver function damage analysis were all also comparable between the two groups.

### Shortened duration of viral shedding in the nelfinavir treated patients

**Fig. 3:**
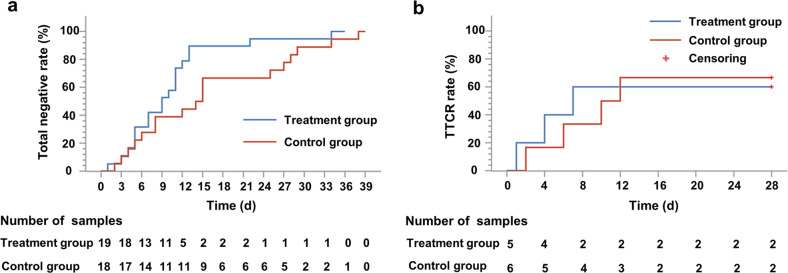
Outcomes over time. **a** Total rate of converting to negative throat swab of SARS-CoV-2; **b** TTCR rate

For the primary endpoint of time to clinical recovery (TTCR), which was the time to convert to negative throat swab of SARS-CoV-2, the nelfinavir group had a 5.5 day shorter median time (9.0 days [IQR 5.0–11.0]) than the control group (14.5 days [6.0–25.0], *p* = 0.055; Supplementary Table S9, Fig. 3a).

Clinical improvement was observed in the nelfinavir group with a shorter TTCR time of 7.0 days (1.0~ NE (Not Evaluable), *N* = 5), while the control group had a longer TTCR time of 11.0 days (2.0~ NE, *N* = 6). No significance between the two groups was observed probably due to the small samples and large variations (Supplementary Table S10, Fig. 3b).

As of the 7th and 14th days of treatment, the proportion of subjects whose respiratory specimens were negative for SARS-CoV-2 RNA was 31.6% and 68.4% in the nelfinavir group (*N* = 19), while 33.3% and 50.0% in the control group (*N* = 18), respectively, without statistic difference between the two groups.

### Clinically insignificant changes of chest CT in the nelfinavir treated patients

On the day 7 of treatment, subjects with mild disease did not progress to pneumonia in either group. On the 14th day of treatment for the chest CT results of moderate COVID-19, one patient was abnormal and had no clinical significance in the nelfinavir group, whereas three individuals in the control group were abnormal and clinically significant, with no discernible difference between the two groups statistically. On the 14th day of treatment for patients with mild COVID-19, there were no subjects in either the nelfinavir group (*N* = 5) or the control group (*N* = 4) progressed to severe or critical pneumonia.

### Significantly shortened the duration of fever time of the nelfinavir treated group

59.5% (22/37) patients had clinically significant abnormal body temperature ranging between 37.1 and 39.2 (Supplementary Table S11, S12). In details, 47.4% (9/19) cases in the nelfinavir group and 72.2% (13/18) cases in the control group have fever. For the patients with fever, the average fever days in the nelfinavir group are 2.8 ± 1.6 days, while the patients in the control group have significantly longer fever time (6.6 ± 4.1 days; *P* = 0.014).

### Nelfinavir therapy observed no serious adverse events in COVID-19 patients

The adverse events occurred during the trial were all grade 1. Among them, 10 patients in the nelfinavir group (*N* = 19) had 15 cases of grade 1 adverse events (52.6%), and 10 patients in the control group had 16 cases of grade 1 adverse events (55.6%). There was no significant difference in the occurrence of adverse events during treatment across groups. No patients died of illness in either group on the day 28.

After treatment, there were no clinically significant patients with abnormal sitting diastolic blood pressure, sitting systolic blood pressure, respiratory rate, and pulse in the two groups.

### Nelfinavir has unchanged inhibitory activity against different SARS-CoV-2 variants

Nelfinavir was determined to have the EC_50_ values of 2.93, 2.09 and 2.03 μM against wild type SARS-CoV-2, Delta and Omicron mutants in Vero E6 cells, while of 1.86, 1.76 and 1.73 μM in Calu-3 cells, respectively (Table 1, Supplementary Fig. S4), showing that the SARS-CoV-2 virus does not acquire resistance to nelfinavir as a result of mutations that occur naturally. As positive controls, remdesivir and paxlovid are also insensitive to the naturally occurring mutations (Table 1, Supplementary Fig. S5, S6).

## Discussion

### Multiple roles of nelfinavir as anti-COVID-19 agent

With virtual screening and molecular dynamics approaches, nelfinavir was predicted to be promising M^pro^ inhibitor.^16–20^ Multiple studies have been conducted on the inhibition of nelfinavir against SARS-CoV-2 M^pro^, with IC_50_ values determined to be from 37 to 600 μM or even inactive.^21–26^ However, we determined the IC_50_ value to be 8.26 μM after a 63-min preincubation, although the IC_50_ value is weaker than 20 μM without preincubation. Therefore, our result indicates that the nelfinavir binding to M^pro^ is a slow association process, which needs long time to fulfill its inhibitory activity. However, the in vitro and in vivo activities of nelfinavir against SARS-CoV-2 cannot be attributed only to its anti-M^pro^ activity, but should be attributed to its multiple target mechanism, e.g., cyclophilin A that is a host-encoded and broad-range anti-coronaviruses target.^27^ It was reported that nelfinavir binds cyclophilin A with a *K*_D_ of 0.94 μM.^28^ The IC_50_ of nelfinavir against cathepsin L was determined to be 18.0 μM.^29^ Nelfinavir could completely inhibit SARS-CoV-2 spike mediated cell fusion at 10 μM.^30^ We also found that the IC_50_ of nelfinavir against SARS-CoV-2 RdRp is 111.80 ± 8.20 μM (Supplementary Fig. S7), and the inhibitor rate at 100 µM against papain-like protease is 33.28 ± 2.47% (data not shown).^15,31^ Nelfinavir has been shown to successfully inhibit inflammatory cytokines at 2.5 μM in vitro, and to suppress inflammatory cytokine in a group of 31 pediatric HIV-1 patients treated for >2 years of therapy.^32^ A clinical trial was found with aim to reposition nelfinavir for treating systemic lupus erythematosus (https://clinicaltrials.gov/ct2/show/NCT02066311). Therefore, nelfinavir might have potential for treating both the virus replication and the virus induced inflammation. The observed reduction in body temperature in the SARS-CoV-2 infected rhesus macaques and patients might be ascribed partially to the anti-inflammation effect of nelfinavir.

### Higher concentration of nelfinavir in lung and bronchoalveolar lavage (BAL) fluid

Nelfinavir have a high concentration in lung. In rats, the lung-to-plasma ratio of nelfinavir is 3.24, detected at 4 h after oral administration.^33^ In mice, we found that the lung-to-plasma ratios of nelfinavir are 2~3 during 0.5 to 9 h after administered orally (Supplementary Table S13). In human, the nelfinavir plasma concentrations are 7.04 and 3.87 μM for *C*_max_ and *C*_trough,Morning_, respectively, at the dosage of 1250 mg BID (VIRACEPT FDA Package Insert Reference ID: 3986049.). In HIV-infected patients, the C_max_ value of nelfinavir in peripheral blood mononuclear cells (PBMCs) was reported to be ~15-fold higher than that of plasma.^34^ In another clinic trial, nelfinavir cellular accumulation in PBMCs was 5.30-fold higher than that in plasma in HIV-infected patients.^35^ The intracellular nelfinavir concentration in infected monkey lungs was roughly estimated to be 0.55 * 3.24 * (5.3~15) = 9.4 ~ 26.7 μM, which is higher than the in vitro antiviral EC_50_ value. Therefore, the in vivo and clinical efficacies could be at least partially attributed to the high distribution of nelfinavir in lungs and in PBMCs.

Other HIV protease inhibitors, i.e., lopinavir-ritonavir, have been shown to have insignificant benefit in COVID-19 patients^36^ and in a ferret infection model.^37^ The insufficiency of lopinavir-ritonavir might be mainly attributed to the undetectable lopinavir-ritonavir in lungs in most patients, where only 16.7% of patients had detectable lopinavir-ritonavir in their bronchoalveolar lavage (BAL) fluid.^38^ Differently, nelfinavir was detectable in 100% of the patients’ BAL fluid and the concentration of nelfinavir in the lung epithelial lining fluid was found to be comparable to that in plasma,^38^ demonstrating nelfinavir’s significant penetration into the alveolar compartment. The lung distribution of nelfinavir in patients is approximately three times as high as that of plasma which is similar to the distribution in lungs of rat,^33^ while the distribution of lopinavir in lungs of rat is 0.51 times as high as that in plasma. Similar to lopinavir-ritonavir, although many drugs and new chemicals have been found to be potent against SARS-CoV-2 at cell level, most of them did not show benefit in COVID-19 patients or infected monkeys.

### Good safety profile to the people at almost all ages and during pregnancy

Nelfinavir was approved for HIV infection in adults and adolescents aged 13 and older (750 mg three times a day with meal or 1250 mg twice a day with meal), and children with age from 2 to <13 years (25 to 35 mg/kg three times a day with meal or 45 to 55 mg/kg twice a day with meal). In addition, nelfinavir was found to be safe in children <2 months old, and between 2 months and 2 years old^39^ and in pregnant patients,^40,41^ even in the first 2 weeks of life,^42^ suggesting the good safety profile of nelfinavir in clinic in almost all ages of children and adults including pregnant women. 3125 mg twice daily was determined to be the maximum tolerated dosage (MTD) in adults with solid tumors,^43^ and MTD was not reached at dose 4250 mg twice daily in liposarcoma.^44^

In summary, nelfinavir could reduce the activity of SARS-CoV-2 main protease and the virus replication in Vero E6, it is also active in suppressing SARS-CoV-2 shedding in the monkeys and can lower viral replication in the lungs by approximately three orders of magnitude when used prophylactically in a rhesus macaque model. In clinic, nelfinavir has the efficacy to shorten the length of viral shedding in COVID-19 individuals with mild/moderate disease by 5.5 days (*P* = 0.055), to shorten the median time for TTCR by 4.0 days (*P* = 0.899), and significantly shorten the duration of fever time. It should be noted that in the clinical investigation, there was no significant difference between the nelfinavir group and the control group in terms of TTCR time and chest CT changes, showing the necessity of larger scale clinical study to clarify the limitation of the current research results. Taking into account its good safety profile in almost all ages and in pregnant, its greater exposure in lung than in plasma, and its potential to reduce inflammation, we believe that nelfinavir is a promising therapeutic candidate as COVID-19 therapy that warrants further investigation in larger clinical trials.

## Materials and methods

### Animal study

#### Ethics and biosafety statement

The Laboratory Animal Center, Kunming Institute of Zoology (KIZ), the Chinese Academy of Sciences (CAS) provided all the 6 rhesus macaques. The animal ethics committee of KIZ, CAS gave its approval (certificate No.: IACUC20016) to all the experiments performed on monkeys in the National Kunming High-Level Biosafety Primate Laboratory Center. The temperature and humidity of the animal’s houses stay the same, and the light goes on and off every 12 h. During the experiment, they are fed twice a day. Water was available *ad libitum*.

### Study design

6 healthy male rhesus macaques (ages 3–6 years) were challenged via a combination of intratracheal (1.2 mL, fiberoptic bronchoscopy) and intranasal (0.4 mL/nostril). The total virus titer was 1 × 10^7^ TCID_50_, dilution with sterile 0.9% saline. Via the injection of Zoletil 50 (Virbac, France) into the thigh muscle, the monkeys were anesthetized, and then the body temperature and weight were measured in sequence; nasal swab, throat swab, anal swab and peripheral venous blood samples were collected in sequence, and heparin was used for venous blood anticoagulation. Detection and sample collection time were 0 day before the challenge and 1, 3, 5, and 7 days after the challenge. On the 8th day after the challenge, euthanasia was carried out, and tissues of heart, liver, spleen, left lower lobe, kidney, stomach, jejunum, colon, bladder, thymus and biceps were collected.

**Fig. 4:**
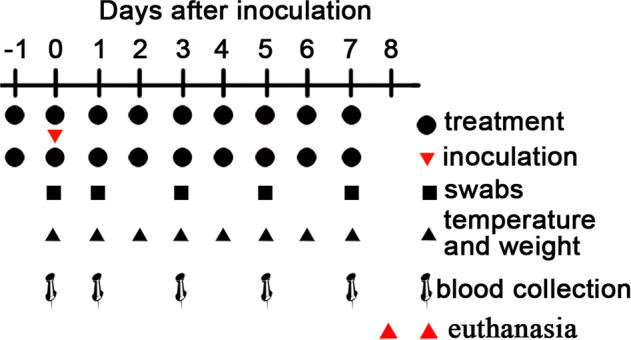
Schedule of nelfinavir treatment and SARS-CoV-2 infection in rhesus macaques. To evaluate the prophylactic efficacy of nelfinavir treatment in the SARS-CoV-2 infection model in rhesus macaques, one group of three rhesus macaques was administered 200 mg/kg nelfinavir three times by nasogastric administration before SARS-CoV-2 (strain 20SF107) inoculation (black circles). One group of three control animals was administered 5 mL/kg vehicle solution (0.5%CMC-Na) by nasogastric administration. Treatment was continued twice daily until 7 dpi, then all animals were euthanized on 8dpi. At 0, 1, 3, 5, and 7 dpi, nasal, throat, and rectal swabs were collected. Temperature and weight changes were monitored at 0, 1, 2, 3, 4, 5, 6, and 7 dpi. Blood samples were taken at 0, 1, 3, 5, and 7 dpi

The rhesus macaques were divided into treatment group (3 males) and control group (3 males). The dosage was based on body weight. The treatment group was 200 mg/kg. The 0.5% CMC-Na suspension (5 mL/kg) was given to the control group. The administration time is 10:00 in the morning and 20:00 in the evening, with an interval of 10 h. The frequency of administration is BID. The mode of administration is gastric administration via a nasogastric tube. Before each administration, 25 ml of apple oatmeal is given in advance, then the medicine is given, and finally 5 ml of apple oatmeal is given. The treatment group was administered 3 times before the challenge, and then 2 times on the 1st to 7th days after the challenge; the control group started 6 h after the challenge, the continuous dosing cycle is once on the 0th day before the challenge, and the 1st to 7th days after the challenge. To quantify the plasma level of nelfinavir, blood samples were collected at 15:00 on days 1, 3, 5 and 7 (Fig. 4). The plasma samples were immediately treated with three volumes of ethanol to kill the virus for further liquid chromatography/mass spectrometry based analysis.

### Lung distribution study in mouse

Male ICR mice were randomly divided into two groups (21 mice each) and given a single oral dosage of nelfinavir mesylate at 250 or 750 mg/kg (via gavage). By bleeding from the orbital sinus, mice under isoflurane anesthesia were killed at 0.5, 1, 3, 6, 9, 12, and 17 h (three mice per time point) after dosing. The blood samples were collected into heparinized tubes, and the plasma fractions were obtained by centrifugation. The mouse’s lungs were removed, cleaned in ice-cold saline, blotted, weighed, and homogenized in four-fold volumes of ice-cold water. In order to preserve the samples for liquid chromatography/mass spectrometry analysis, the mouse plasma and lung homogenate samples were kept at −70 °C.

### LC-MS-based bioanalytical assays

The amount of nelfinavir in mouse lung homogenate and plasma was measured using an AB Sciex API 4000 Q Trap mass spectrometer (Toronto, Canada), interfaced with a Turbo V ion source and an Agilent 1290 Infinity II LC (Waldbronn, Germany). Ten volumes of methanol (three volumes of ethanol was used instead for rhesus macaques plasma samples obtained from SARS-CoV-2 model) were used to precipitate the samples, followed by a further centrifugation. With a 5 μm Agilent ZORBAX Eclipse Plus C18 column (50 mm × 2.1 mm i.d.; Santa Clara, CA, USA), chromatographic separation was accomplished using a mobile phase (delivered at 0.3 mL/min) that contained (A) water (containing 9 mmol/L formic acid) and (B) acetonitrile (containing 9 mmol/L formic acid). A 6-min gradient program that included 0–1.0 min, at 10% B; 1.1–2.5 min, at 65% B; 2.6–4.0 min, at 100% B; 8.5–10 min, at 100% B; and 4.1–6.0 min at 10% B was employed. The positive ion mode of operation was used for the mass spectrometer. Optimal precursor-to-product ion pairs for multiple reaction monitoring of nelfinavir and the internal standard reserpine were m/z 568 → 330 (collision energy, 45 V) and 609 → 195 (48 V), respectively. Weighted (1/X) linear regression of the peak areas (Y) of the analytes against the corresponding nominal analytes’ concentrations (X; 4.12, 12.4, 37.0, 111, 333, 1000, and 3000 nmol/L) was used to construct matrix-matched calibration curves with reference standards, and the curves demonstrated strong linearity (*R* > 0.99).

### Virus strain and cell

The virus used in the experiment was the SARS-CoV-2 strain 20SF107, provided by the Guangdong Provincial Center for Disease Control and Prevention, Guangdong, China. In Vero-E6 cells, the viral strain was amplified, and the Median tissue culture infective dose (TCID_50_) was estimated by the Reed-Muench method.^45^

### Sample tests

SARS-CoV-2 RNA was detected by Quantative real-time RT-PCR (qRT-PCR). Total RNA from swabs was extracted using a High Pure Viral RNA Kit (Roche, Germany) according to the manufacturer’s instructions, and RNA of tissues were isolated by TRIzol reagent method (Thermo USA).^46^ A probe one-step real-time quantitative PCR kit (TOYOBO, Japan) was used to detect viral RNA. Previously described primers targeting the N protein and probe were employed, including (5'-GGGGAACTTCTCCTGCTAGAAT-3', 5'-CAGACATTTTGCTCTCAAGCTG-3', FAM-TTGCTGCTGCTTGACAGATT-TAMRA-3').^47^ Each test run’s dilution refers to the standard (National Institute of Metrology, China). Lastly, each sample’s copy number is computed. After the SARS-CoV-2 challenge, the plasma was separated from peripheral blood at 1, 3, 5, and 7 days.

#### Statistical analysis

R version 3.3.3 was used to perform statistical analyses. Two-way mixed ANOVA was used to compare the mean differences (temperature, weight, virus loads, etc.) between groups that were divided into two “factors” (independent variables), one of which is a “within-subjects” factor (time) and the other is a “between-subjects” factor (treatment or control). For anal swabs, there is only one none-zero data points in the treatment group. Therefore, the ANOVA method relying on variance could not be used, and Mann-Whitney U test was used instead. For viral loads in tissues, *t*-test was used.

### Clinical trial

#### Study design

It was an investigator-initiated prospective, individually randomized, open-label, single-center trial to evaluate the efficacy and safety of oral nelfinavir in adults (aged ≥ 18 years) with mild or moderate COVID-19. The study was carried out at Shanghai Public Health Clinical Center in Shanghai, China. The ethics committee of Shanghai Public Health Clinical Center granted ethical approval on Jun 2, 2020. This clinical trial was done according to the principles of the *Declaration of Helsinki* (2013) and *Good Practice for Clinical Trials of Drugs* (Revised in 2020). Both the protocols are available online. Although nelfinavir could be administrated orally 750 mg (×3 times daily) or 1250 mg (×2 times daily), we decided to use the later for this study as the bioavailability of nelfinavir increased in healthy human volunteers more rapidly than its dosage (AUC_0-∞_ of 1250 ± 230 ng·h/mL at the dosage of 100 mg *vs*. AUC_0-∞_ of 22208 ± 6306 ng·h/mL at the dosage of 800 mg).^48^

### Patients

COVID-19 virologically verified adult patients admitted to hospital between Jul. 14, 2020 and Apr. 2, 2021, were recruited from Shanghai Public Health Clinical Center. Eligibility criteria for the trial were RT-PCR positive for SARS-CoV-2, men and non-pregnant women, age at least 18 years. Patients were diagnosed as mild or moderate COVID-19 cases based on the *Diagnosis and Treatment Protocol for Novel Coronavirus Pneumonia* (Trial Version 7 or 8). Mild case means the clinical symptoms were mild, and the imaging revealed no evidence of pneumonia. Moderate case means the patient showed fever and respiratory symptoms and pneumonia on radiological examination. Exclusion criteria included: any situations where the clinical trial cannot be carried out safely; allergies to 2 or more different foods/drugs, or allergies to nelfinavir and its excipients; accompanied by serious diseases that affect survival; having moderate to severe liver injury (Child-Pugh B or C, score ≥ 7); pregnancy or breast feeding; transferability to a non-study hospital in 72 h; have been continuously using immunosuppressive agents or organ transplants in the past 6 months; critical cases diagnosed by the *Diagnosis and Treatment Protocol for Novel Coronavirus Pneumonia* (Trial Version 7 or 8); cannot collaborate in their mental state, suffer from mental disease, lack self-control, and are unable to explain themselves clearly; with HIV; in the acute and active phase of HBV or HCV infection; participating in other clinical trials.

### Randomization and masking

Patients were assigned at random to either the nelfinavir treatment group or the control group, in the ratio of 1:1, by simple randomization. Randomized treatment was open-label.

### Procedures

In the treatment group, patients orally received 1250 mg nelfinavir (625 mg per tablet, provided by Agouron Pharmaceuticals, LLC, a subsidiary of Pfizer Inc.) every 12 h for 14 days, or according to the investigator’s judgment, the patient can continue to take nelfinavir until the efficacy endpoint is reached or the investigator judges that the patient cannot benefit from nelfinavir. Patients assigned to the control group received only basic treatment.

### Clinical and laboratory monitoring

Within 3 days after the last dosing, safety follow-up of subjects (including subjects who withdrew early) was completed, including: physical examination, vital signs collection, nucleic acid test of SARS-CoV-2, laboratory examination, 12-lead electrocardiogram examination, chest CT, blood gas analysis, pneumonia severity (PSI) examination, follow-up of adverse events, severe adverse events, and collection of combination therapy information.

### Outcomes

The time that took for a throat swab test for SARS-CoV-2 to become negative was the primary endpoint. The secondary endpoint is the time to clinical recovery (TTCR), defined by the time from the beginning of treatment to that the respiratory rate, fever, blood oxygen saturation become normal, and the cough is relieved, which lasts for at least 72 h. Fever is defined as oral temperature ≥37.1 °C, or axillary temperature ≥36.6 °C, or rectal temperature or ear temperature ≥37.8 °C. A normal respiratory rate is ≤24 times per minute (under indoor air). A normal blood oxygen saturation is >94% (under indoor air). Cough relief means the patient’s cough must be mild or no, according to the classification of “severe/moderate/mild/none”. Severe is frequent coughing, which seriously affects daytime life or seriously affects night sleep; moderate is also frequent coughing, but lightly affecting daytime life or lightly affecting night sleep; mild is occasional short-term cough or occasional night cough; none is no cough.

### Statistical analysis

Full analysis set (FAS) is the collection of all randomized individuals who received treatment at least once. A per-protocol set (PPS) is a data set generated by participants who are completely compliant with the trial protocol, such as the treatment received, the availability of primary endpoint measures, and the absence of significant deviations from the trial protocol. The primary efficacy measure was determined via PPS analysis. Safety data set (SS) is the actual data on at least one treatment with recorded safety indicators after treatment.

Statistical analysis was performed using SAS 9.4 software. All statistical tests were two-sided, and statistical significance for the differences investigated was defined as a *P*-value ≤0.05. Mean, standard deviation, median, minimum and maximum values were used to define continuous variables. The frequency and percentage of counts and grades were used to characterize the data. R*C chi-square was used to compare differences between groups for Chest CT on day 14 of treatment.

## Supplementary information

Supplemental Material

## Data Availability

The data are available from the corresponding authors upon reasonable request.
