# Supplementary material for: Preventive and therapeutic benefits of nelfinavir in rhesus macaques and human beings infected with SARS-CoV-2
Source: Signal Transduct Target Ther. 2023 Apr 24;8:169. doi: 10.1038/s41392-023-01429-0 (PMC10123561; doi:10.1038/s41392-023-01429-0)
Supplement: Supplementary file 1 — Supplemental Material [file 41392_2023_1429_MOESM1_ESM.docx]

Supplementary Materials for

Preventive and therapeutic benefits of nelfinavir in rhesus macaques and human beings infected with SARS-CoV-2

Zhijian Xu, Danrong Shi, Jian-Bao Han, Yun Ling, Xiangrui Jiang, Xiangyun Lu, Chuan Li, Likun Gong, Guangbo Ge, Yani Zhang, Yi Zang, Tian-Zhang Song, Xiao-Li Feng, Ren-Rong Tian, Jia Ji, Miaojin Zhu, Nanping Wu, Chunhui Wu, Zhen Wang, Yechun Xu, Cheng Peng, Min Zheng, Junling Yang, Feifei Du, Junliang Wu, Peipei Wang, Jingshan Shen, Jianliang Zhang, Yong-Tang Zheng, Hangping Yao, and Weiliang Zhu

Correspondence to: Jingshan Shen, shenjingshan@simm.ac.cn; Jianliang Zhang, zhangjianliang@shphc.org.cn; Yong-Tang Zheng, zhengyt@mail.kiz.ac.cn; Hangping Yao, yaohangping@zju.edu.cn; Weiliang Zhu, wlzhu@simm.ac.cn

**This PDF file includes:**

Materials and Methods

Supplementary Text

Figures. S1 to S7

Tables S1 to S13

References

**Materials and Methods**

**1. *In silico* studies.**

**1.1 Homology modelling.**

43 M^pro^ complexes with ligands were downloaded from protein data bank (PDB IDs: 1WOF, 2A5I, 2A5K, 2ALV, 2AMD, 2GTB, 2GX4, 2GZ7, 2GZ8, 2OP9, 2QIQ, 2V6N, 2ZU4, 2ZU5, 3SN8, 3SND, 3SZN, 3TIT, 3TIU, 3TNS, 3TNT, 3V3M, 4F49, 4MDS, 4TWW, 4TWY, 4WY3, 4YLU, 4YOG, 4YOI, 4YOJ, 4ZRO, 5C5N, 5C5O, 5EU8, 5N5O, 5N19, 5NH0, 5WKJ, 5WKK, 5WKL, 5WKM, 6FV1) and aligned to 2GTB in PyMOL.^1^ 11 complexes (PDB IDs: 2A5K, 2GTB, 2GX4, 3SND, 3TNS, 3V3M, 4F49, 4YLU, 5NH0, 5WKJ, 5WKM) were served as templates to build 11 SARS-CoV-2 M^pro^ models in SWISS-MODEL server by “user template” mode.^2^

**1.2. Approved Drugs.**

1905 approved small molecule drugs with 3D coordinates were downloaded from DrugBank release version 5.1.5,^3^ while 1903 drugs could be converted to pdbqt format by prepare_ligand4.py script in MGLToos version 1.5.6.^4^

**1.3. Molecular Docking.**

1903 approved drugs in pdbqt format were docked to SARS-CoV-2 M^pro^ model (template: 2GTB) by smina,^5^ which is a fork of AutoDock Vina^6^ with improving scoring and minimization. The hydrogens were added to SARS-CoV-2 M^pro^ model by pdb2pqr (--ff=amber --ffout=amber --chain --with-ph=7).^7^ Then the model was converted to pdbqt format by prepare_receptor4.py script in MGLToos version 1.5.6.^4^ The ligand in 2GTB was used to define the grid and the buffer space was set to 6.0 Å (autobox_add). The random seed was explicitly set to 0 (seed). The exhaustiveness of the global search was set to 32 (exhaustiveness) and at most 1 binding mode was generated for each drug (num_modes). MolShaCS, which utilized Gaussian-based description of molecular shape and charge distribution, was used to calculate the 3D similarities between approved drugs and available M^pro^ inhibitors.^8^ Using the same docking parameters, nelfinavir was docked to SARS-CoV-2 M^pro^ (PDB ID: 7VH8).

**1.4. Molecular dynamics simulation.**

Each simulation system was immersed in a cubic box of TIP3P water that was extended by 9 Å from the solute, with a rational number of counter ions of Na^+^ or Cl^-^ to neutralize the system. General Amber force field (GAFF)^9^ and Amber ff03 force field^10^ were used to parameterize the ligand and protein, respectively. 10,000 steps of minimization with constraints (10 kcal/mol/Å^2^) on heavy atoms of complex, including 5,000 steps of steepest descent minimization and 5,000 steps of conjugate gradient minimization, was used to optimize each system. Then each system was heated to 300 K within 0.2 ns followed by 0.1 ns equilibration in NPT ensemble. Finally, 5 ns MD simulation on each system at 300 K was performed. The minimization, heating and equilibrium are performed with *sander* program in Amber16. The 5 ns production run was performed with *pmemd.cuda*.

**1.5. Binding free energy calculation.**

Based on the 5 ns MD simulation trajectory, bind free energy (Δ*G*) was calculated with MM/GBSA^11,12^ and SIE^13^ approaches. In the MM/GBSA, the Δ*G* was calculated according to equation (1),

$\Delta G=\Delta H-T\Delta S=\Delta E_{ele}+\Delta E_{VDW}+\Delta G_{gb}+\Delta G_{np}-T\Delta S$(1)

where Δ*E*_ele_ and Δ*E*_VDW_ refer to electrostatic and van der Waals energy terms, respectively. Δ*G*_gb_ and Δ*G*_np_ refer to polar and non-polar solvation free energies, respectively. Conformational entropy (*T*Δ*S*) was calculated by *nmode* module in Amber16. The dielectric constants for solvent and solute were set to 80.0 and 1.0, respectively, and OBC solvation model (igb = 5 and PBradii = 5)^14^ was used in this study. Other parameters are set to default values.

In the SIE, the Δ*G* was calculated based on equation (2),

$\Delta G_{bind}=\alpha\left[ E_{C}\left( D_{in} \right)+\Delta G_{bind}^{R}\left( \rho,D_{in} \right)+E_{VDW}+\gamma\Delta MSA\left( \rho\right) \right]+C$ (2)

where *E*_C_ and *E*_VDW_ refer to the sum of intermolecular Coulomb and van der Waals interaction energies, respectively. Δ*G*^R^_bind_ and Δ*MSA* refer to the changes of reaction field energy and molecular surface area upon ligand binding, respectively. Default values of the global proportionality coefficient (*α* = 0.1048), the solute interior dielectric constant (*D_in_* = 2.25), the van der Waals radii linear scaling coefficient (*ρ* = 1.1), the molecular surface area coefficient (*γ* = 0.0129 kcal/mol/Å^2^), and the constant (*C* = -2.89 kcal/mol) are used in this study.

**2. M^pro^ Enzyme Assay**

**2.1 Chemicals and reagents**

SARS-CoV-2 M^pro^ was expressed and purified according to previous study.^15,16^ The Smt3-SARS-CoV-2 M^pro^ codon was cloned into the pET29a (+) vectors by GENEWIZ, Inc. (Beijing, China). Escherichia coli (E. coli) BL21 (DE3) was gained from Shanghai Weidi Biotechnology Co., Ltd. (Shanghai, China). Potassium hydrogen phosphate anhydrous (K_2_HPO_4_) and Potassium dihydrogen phosphate (KH_2_PO_4_) were purchased from Sinopharm Chemical Reagent Co., Ltd. (Shanghai, China). Ethylene Diamine Tetraacetic Acid (EDTA) was gained from Dalian Meilun Biotechnology Co. Ltd. (Dalian, China). Fluorescent substrate (Dabcyl-KNSTLQSGLRKE-Edans) was purchased from Shanghai Sangon Biological Engineering & Technology and Service Co. Ltd. (Shanghai, China), with the purity of 99%. Nelfinavir Mesylate (nelfinavir, CAS: 159989-65-8, Formula: C_33_H_49_N_3_O_7_S_2_, MW: 663.89) was purchased from TargetMol with the purity of 98.3%.

**2.2 Expression and purification of SARS-CoV-2 M^pro^**

According to published research, an expression vector of SARS-CoV-2 M^pro^ for *E. coli* BL21 (DE3) was generated.^15^ Concisely, the SARS-CoV-2 M^pro^ recombinant expression plasmid was designed utilizing pET29a(+) to link the protein Smt3 and SAVLQ at the N-terminus, and the HRV 3C protease cleavage site (SGVTFQGP) conjugated to a His6-tag at the C-terminus. SARS-CoV-2 M^pro^ was expressed using the previously described auto-induction method.^16^ The cells were lysed in ice by sonication, and the lysate was centrifuged for 30 min at 4°C and 18000 rpm. The supernatant was added to 2 mL of Ni-NTA agarose (GE Healthcare), eluted with 300 mM imidazole and then separated using Superdex 200 10/300 GL column (GE Healthcare). For the following inhibitory assay, the protein of interest was condensed using a 10 kDa molecular weight cut-off (MWCO) concentrator and kept in buffer (25 mM HEPES, 150 mM NaCl, 1 mM DTT, pH 7.4).

**2.3** **SARS-CoV-2 M^pro^ inhibition assay**

The inhibitory activity of nelfinavir was determined using the previously developed enzyme inhibition assay based on fluorescence resonance energy transfer (FRET).^17,18^ In a mixture of 200 μL, the SARS-CoV-2 M^pro^-mediated reaction was performed carried out. Concisely, the SARS-CoV-2 M^pro^ (2 μg/mL) was preincubated with nelfinavir or DMSO in PBS (pH 7.4, 100 mM, 1 mM EDTA) for 3 min or 63 min at 37 °C. Then, the hydrolysis reaction was proceeded for 20 min after the addition of Dabcyl-KNSTLQSGLRKE-Edans (20 μM) to the mixture. Finally, the microplate reader (SpectraMax® iD3, Molecular Devices, Austria) was used to measure the excited fluorescence (excitation / emission, 340 nm / 490 nm).

**3. Antiviral assay**

**3.1 Materials**

The Vero E6 (African green monkey kidney, ATCC^®^ CRL-1586^TM^) cell line and Calu-3 (Human lung adenocarcinoma, ATCC^®^ HTB-55 ^TM^) cell line were purchased from ATCC and kept in the State Key Laboratory for Diagnosis and Treatment of Infectious Diseases. SARS-CoV-2 was isolated from patients’ samples and validated by sequence analysis of the complete genome (hCoV-19/Hangzhou/ZJU-05/2020, GISAID Accession ID: EPI_ISL_415711^19^; hCoV-19/Hangzhou/ZJU-12/2020, GISAID Accession ID: EPI_ISL_3127444.2, hCoV-19/Zhejiang/ZJU-15/2022, GISAID Accession ID: EPI_ISL_16468827). For viral RNA extraction and test, Magnetic Viral RNA Extraction kit (MVR01) and quantitative real-time RT-PCR (qRT-PCR) kit were purchased from Liferiver. Cell Counting Kit-8 (CCK-8) was purchased from DojinDo to test cell proliferation and cytotoxicity. Fetal Bovine Serum (FBS) was purchased from Life Technologies. MEM Medium was purchased from Life Technologies. Penicillin and Streptomycin were purchased from Life Technologies. TPCK-Trypsin (from bovine pancreas Type VIII) was purchased from Life Technologies. Disposable experimental consumables, e.g., cell culture plates, were purchased from Corning. Body protective coverings for BSL-3 laboratories were purchased from 3M Company. CO_2_ incubator (Thermo 3110) was purchased from Thermo. ZOE Fluorescent Cell Imager was purchased from BioRad. Real-Time PCR detection system Bio-Rad CFX96 and Microplate Reader Bio-Rad 680 were purchased from BioRad. Biological safety cabin (BSC) was purchased from Thermo. Tabletop refrigerated high speed centrifuges was purchased from Thermo. Pipettes was purchased from Eppendorf. Electronic balance was purchased from Mettler Toledo. All experiments involving infectious virus were conducted in a China National Accreditation Service for Conformity Assessment (CNAS) approved biosafety level III laboratory, State Key Laboratory for Diagnosis and Treatment of Infectious Diseases in Zhejiang University.

**3.2 drug solution preparation.**

10mM Nelfinavir Mesylate, Remdesivir, and Nirmatrelvir was stored in DMSO, -20°C and diluted in MEM to the concentrations of 100, 50, 25, 12.5, 6.25, 3.12, 1.26 and 0.78 μM, respectively.

**3.3 Cytotoxicity of Nelfinavir Mesylate and other drugs on Vero E6 cells.**

**Cell proliferation and cytotoxicity assay (CCK-8):**

(1) Vero E6 cells were plated at 5000 cells per well in a 96-well plate and incubated at 37°C in 5% CO2 incubator. After the formation of a monolayer, wash the cells two times with Hanks' BSS.

(2) Drug dilution: Nelfinavir Mesylate and other drugs were diluted in virus growth medium (500 mL MEM with 2% FBS, 100U/mL penicillin, 100 μg/mL streptomycin, and 16 μg/mL TPCK-Trypsin). The initial concentration was 100 μM, with eight half-log_10_ dilutions to 0.78 μM. Each drug concentration was in duplicates. 150 μL drugs were added to each well and some wells were used as control. The cells were incubated at 35°C in 5% CO_2_ incubator for 48 hours.

(3) 15 μL CCK-8 solution was added to each well, after 3 hours of incubation, absorbance was measured at 450 nm with a microplate reader, and the cytotoxicity was calculated accordingly.

**3.4 TCID_50_ determination of SARS-CoV-2**.

TCID_50_ is the tissue culture infectious dose defined as the virus dilution needed to infect 50% of the cell monolayers.

**Micro cytopathic inhibition assay:**

(1) Seed 10000 Vero E6 cells or Calu-3 cells per well of 96-well plates. Once cells have been seeded, allow the cells to grow at 37°C in 5% CO_2_ incubator until the cells are evenly distributed and reached over 75-90% confluency. Then discard the cell culture and wash the cells two times with Hanks' BSS (to remove the residue fetal bovine serum (FBS)).

(2) 1000 times dilution of the SARS-CoV-2 isolate by virus growth medium was used as initial concentration. Then make 8 series of dilutions at 1:10 of the initial concentration. 100 μL virus dilution at varying concentrations were added to 4 wells, and the virus growth medium without virus were used as control. The cells were incubated at 35°C in 5% CO_2_ incubator for 6 days.

(3) The cell morphology and CPE effect was observed everyday by the inverted microscope. <25% CPE was defined as ‘+’, 26%-50% CPE as ‘++’, 51%-75% CPE as ‘+++’, and 76%-100% CPE as ‘++++’. The TCID_50_ was calculated by Reed-Muench method.

**3.5 The inhibitory effect of Nelfinavir Mesylate and other drugs against wild type and two variants of SARS-CoV-2 in vitro.**

(1) Cell culture: Seed 50000 Vero E6 cells or Calu-3 cells per well of 24-well plates. Once cells have been seeded, allow the cells to grow at 37°C in 5% CO_2_ incubator until the cells are evenly distributed and reached over 75-90% confluency. Then discard the cell culture and wash the cells two times with Hanks' BSS (to remove the residue fetal bovine serum (FBS)).

(2) Drug and virus dilution: Nelfinavir Mesylate and other drugs was diluted in virus growth medium to the concentrations of 25, 12.5, 6.25, 3.12, 1.26 and 0.78 μM, respectively. Nirmatrelvir was mixed with ritonavir so that the weight ratio is 3:1, the same as for oral use.

(3) Test the antiviral activity on Vero E6 and Calu-3 cells:

① SARS-CoV-2 was propagated in Vero E6 and Calu-3 cells: Add 250 μL virus dilution (200 TCID_50_) per well and the wells without virus or without drugs were used as control. The cells were incubated at 35°C in 5% CO_2_ incubator for 3 hours. Then discard the cell culture containing virus and wash the cells two times with Hanks' BSS.

② Drug treatment: Add 1 mL Nelfinavir Mesylate and other drugs dilution at varying concentrations per well to the Vero E6 cells pre-exposure to virus. Each dilution was in triplication. The cells were incubated at 35°C in 5% CO_2_ incubator for 48 hours.

③ Viral RNA extraction and quantitative real-time RT-PCR (qRT-PCR).

200 μL cell culture supernatant was collected for viral RNA extraction using the Magnetic Viral RNA Extraction kit (MVR01) and Automated Nucleic Acid Extractor (EX3600, from Liferiver) and the elution volume was 50 μL. SARS-CoV-2 RNA was quantified by qRT-PCR using the kit (Liferiver Cat no. Z-RR-0479-02-50) on 5 μL elution.

The number of cycles required for the fluorescent signal to cross the threshold is defined as the Ct (cycle threshold). The lower the Ct level the greater the amount of SARS-CoV-2 RNA in the sample. y=-3.33x+48.69, where y is the Ct value and x is the RNA copy number in log10 value.

**4. SARS-CoV-2 PL^pro^ inhibition assay**

A continuous 10 points fluorometric assay for 5 min was used to assess the activity of SARS-CoV-2 PL^pro^. Briefly, each compound was mixed with the recombinant SARS-CoV-2 PL^pro^ (40 nM at a final concentration) in 50 μL assay buffer (20 mM Tris pH8.0, 0.01% Tween20, 0.5 mM DTT) and incubated for 10 min. The reaction was initiated by adding a final concentration of 50 μM of the substrate Z-RLRGG-AMC (GL Biochem, Shanghai). Excitation and emission wavelengths of 355 nm and 460 nm were measured using an EnVision multimode plate reader (Perkin Elmer, USA).

**5.** **SARS-CoV-2 RdRp inhibition assay**

The detection of SARS-CoV-2 RdRp complex RNA synthesis was established using a real-time assay with the QuantiFluor® dsDNA Dye (Promega), which contains a fluorescent DNA-binding dye that allows for the sensitive quantitation of tiny amounts of double-stranded DNA (dsDNA) in solution. The fluorescence was measured using an EnVision multimode plate reader at wavelengths of 504 nm and 531 nm for excitation and emission (Perkin Elmer, USA).The assay records the dsRNA synthesis in a reaction employing a poly-U template–primer RNA with the sequences of 5’-biotin-UUUUUUUUUUUUUUUUUUUUUUUUUUUUUUAACAGGUUCUAGAACCUGUU -3’ as a template (Sangon Biotech,Shanghai China) and ATP as the nucleotide substrate. Individual wells of white 384-well low volume round bottom plates were used for the reactions. 50 mM Tris-HCl, pH 7.5, 50 mM Ammonium acetate, 0.5 mM MnCl2, 20 μM ATP, 0.2 μM poly-U template–primer RNA, 0.01% Tween-20 were used in the reaction.

**Supplementary Text**

**1. *In silico* prediction of nelfinavir as inhibitor of SARS-CoV-2 main protease (M^pro^)**

*In silico* prediction of the SARS-CoV-2 main protease (M^pro^) inhibitor was performed by docking 1903 approved small molecule drugs to a homology model built with SWISS-MODEL using the crystal structure of 2GTB as template. There are 672 drugs with the docking score better than -7.0 kcal/mol. The binding modes are essential to the activities. If a compound shares a similar binding mode to a known ligand, it is more likely to have a similar activity to the ligand. Therefore, we calculated the 3D similarities of the binding mode of the docked drugs to the available binders against M^pro^. 39 binders in 44 complexes were chosen as the references to calculate the 3D similarities (Table S1). 159 drugs have a 3D similarity larger than 65.0% with at least one known M^pro^ binder.

After visualizing the docked complexes carefully, taking into account both the docking scores and the binding modes similar to that between other 43 viral M^pro^s and their ligands, we selected 15 drugs (Table S2) for further analysis. There are some differences between the conformations of the protein in the 3D similarity reference and 2GTB model. Therefore, we modelled 10 additional homology models using the proteins in 3D similarity reference as templates and we re-docked the 15 drugs to the 10 new homology models. 6 drugs (nelfinavir, pitavastatin, perampanel, praziquantel, zopiclone, and eszopiclone) show good docking scores and binding modes (Table S3). Because eszopiclone and zopiclone were used to treat insomnia in a low dosage which may not be suitable to treat pneumonia, we carried out further binding free energy calculation for the rest 4 drugs (nelfinavir, pitavastatin, perampanel and praziquantel).

**2. Binding Free Energy calculated by MM/GBSA and SIE.**

The 4 docked complex structures, i.e., nelfinavir-2GX4, pitavastatin-2GTB, perampanel-5NH0 and praziquantel-3V3M, were subjected to 5 ns molecular dynamics simulations using Amber 16. To provide insight into their binding mechanisms, the binding free energies were calculated by MM/GBSA and SIE approaches. In results of the MM/GBSA approach, the calculated binding free energies of nelfinavir-2GX4, pitavastatin-2GTB, perampanel-5NH0, and praziquantel-3V3M, are -24.69±0.52, -12.70±0.38, -14.98±0.34, and -6.51±0.21 kcal/mol, respectively (Table S4), which highlight nelfinavir as the most promising one. In pitavastatin-2GTB, perampanel-5NH0, and praziquantel-3V3M, the van der Waals interaction (*E*_vdw_) makes a more significant contribution than the electrostatic interaction (*E*_ele_) (Table S4), indicating that van der Waals interaction is the main driving force for the 3 drugs binding. However, the *E*_ele_ interaction of nelfinavir-2GX4 is very strong, suggesting that the electrostatic interaction also play an important role in the binding of nelfinavir. Furthermore, in results of the SIE approach, the rank of the calculated binding free energies for the 4 docking complexes is consistent with that of the MM/GBSA approach (Table S5) that the nelfinavir has the strongest binding free energy (-9.42±0.04 kcal/mol), indicating the reliability of the current binding free energy analysis. In addition, nelfinavir binds to the stable region of the pocket determined from D3Pockets.^20^

**3. Binding modes of nelfinavir against SARS-CoV-2 M^pro^.**

As shown in Fig. S2, the binding modes of nelfinavir in its docking complex turned out to be very similar with that of the original ligand (TG-0205221)^21^ of 2GX4 (Fig. S2a&b), which is an inhibitor of SARS M^pro^ with in vitro K_i_ of 0.053 μM and IC_50_ of 0.6 μM in the Vero-E6 cells.^21^ In its crystal structure, TG-0205221 is able to form hydrogen bonds with HIS-163, GLU-166 and GLN-189, and a very week hydrogen bond with PHE-140 (Fig. S2c). Our docking results showed that three of the hydrogen bonds involving GLU-166 and GLN-189 maintained upon the binding of nelfinavir and SARS-CoV-2 M^pro^, with additionally the possible formation of π-π stacking interaction with HIS-41 (Fig. S2d). These observations further demonstrated that nelfinavir would interact with key residues of SARS-CoV-2 M^pro^ in a similar way to that of the existing inhibitors against coronavirus M^pro^. When docked to SARS-CoV-2 M^pro^ crystal structure (PDB ID: 7VH8), similar results were yielded with the docking score of -8.25 kcal/mol (Fig. S3a).

**4. Inhibition of** **nelfinavir against SARS-CoV-2 M^pro^**

The SARS-CoV-2 M^pro^ was expressed and purified from *Escherichia coli* BL21 (DE3). Fluorescent substrate (Dabcyl-KNSTLQSGLRKE-Edans) was used for the enzymatic assay. It was found that nelfinavir could inhibit the M^pro^ not only in a dose- but also in a time-dependent manner, with IC_50_ values of 23.04±1.02 μM (3-min incubation) and 8.26±1.04 μM (63-min incubation) (Fig S3b), respectively, suggesting that the inhibition potency would be enhanced over time.

**5. Anti-SARS-CoV-2 activity of nelfinavir in Vero E6 and Calu-3 cells**

The inhibitory activity of nelfinavir against SARS-CoV-2 was carried out in Vero E6 cells. The half-maximal effective concentration (EC_50_) of nelfinavir against the wild type virus was determined to be 2.93 μM (Fig. S4), while the half-cytotoxic concentration (CC_50_) of nelfinavir against Vero E6 cells was determined by CCK-8 assays to be 51.55 μM (Fig. S1). Accordingly, the selectivity index (SI) was estimated to be 17.59.

**6. Details of the nelfinavir-treated and control groups in clinical trial.**

In FAS, the mean age of the nelfinavir group and the control group were 39.2±9.11 and 37.7±15.24, respectively. The median age was 38.0 and 34.0, the maximum age was 62 and 66, and the minimum age was 25 and 19, respectively. There was no significant difference in age between the two groups (*P*=0.723, Table S7). The average weights of the nelfinavir group and the control group were 67.42±11.49 kg and 62.47±6.85 kg, respectively. The median weights were 64.50 kg and 60.00 kg, the maximum weights were 93 kg and 80 kg, and the minimum weights were 54 kg and 55 kg, respectively. There was no significant difference in body weight between the two groups (*P*=0.143, Table S7). There were 13 (68.4%, 13/19) male subjects and 6 (31.6%, 6/19) female subjects in the nelfinavir group. There were 8 (44.4%, 8/18) male subjects and 10 (55.6%, 10/18) female subjects in the control group. There was no significant difference in gender distribution between the two groups (*P*=0.191, Table S7). There were 18 Han Chinese (94.7%, 18/19) in nelfinavir group, 16 Han Chinese (88.9%, 16/18) in the control group, and there was no statistical difference between the two groups (*P*=0.604, Table S7). There were 14 (73.7%, 14/19) cases of mild patients in nelfinavir group, 14 (77.8%, 14/18) in the control group, and 5 (26.3%, 5/19) cases of patients in nelfinavir group, 4 (22.2%, 4/18) in the control group. There was no significant difference in disease severity between the two groups (*P*>0.999, Table S7). The past medical history was comparable between the two groups (Table S8). Before treatment, the history of food and drug allergy, comorbidities and treatment history, physical examination, vital signs, SARS-CoV-2 RNA detection, SARS-CoV-2 IgG and IgM antibody detection, infectious disease serological examination, 12-lead electrocardiogram, TTCR assessment, chest CT examination, and liver function damage analysis were all comparable between the two groups (*P*>0.05).

**7. Multiple roles of nelfinavir as anti-COVID-19 agent**

At 100 μM, nelfinavir showed weak inhibitory activity against PL^pro^，with the inhibition rate of 33.28±2.47%. The IC_50_ of nelfinavir against RdRp is 111.80 ± 8.20 μM.


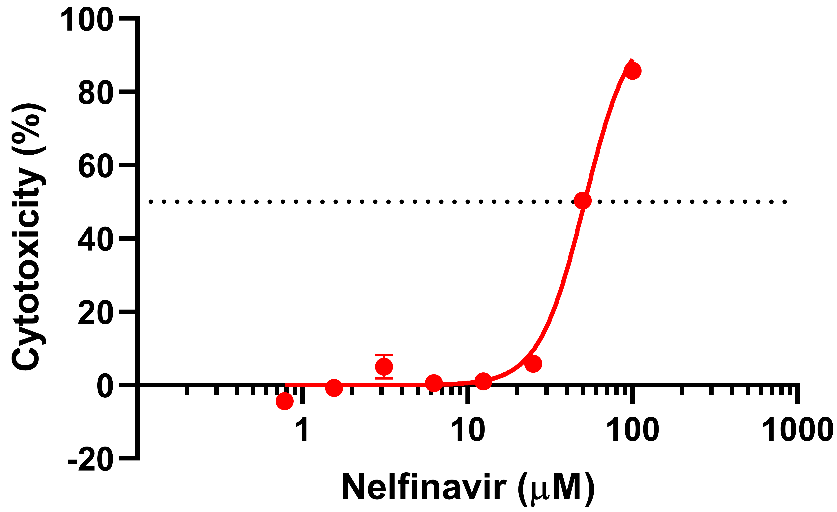


**Figure. S1.** Cytotoxicity of nelfinavir against Vero E6 cells. The Y-axis represents mean cytotoxicity of the drug (in duplicates).


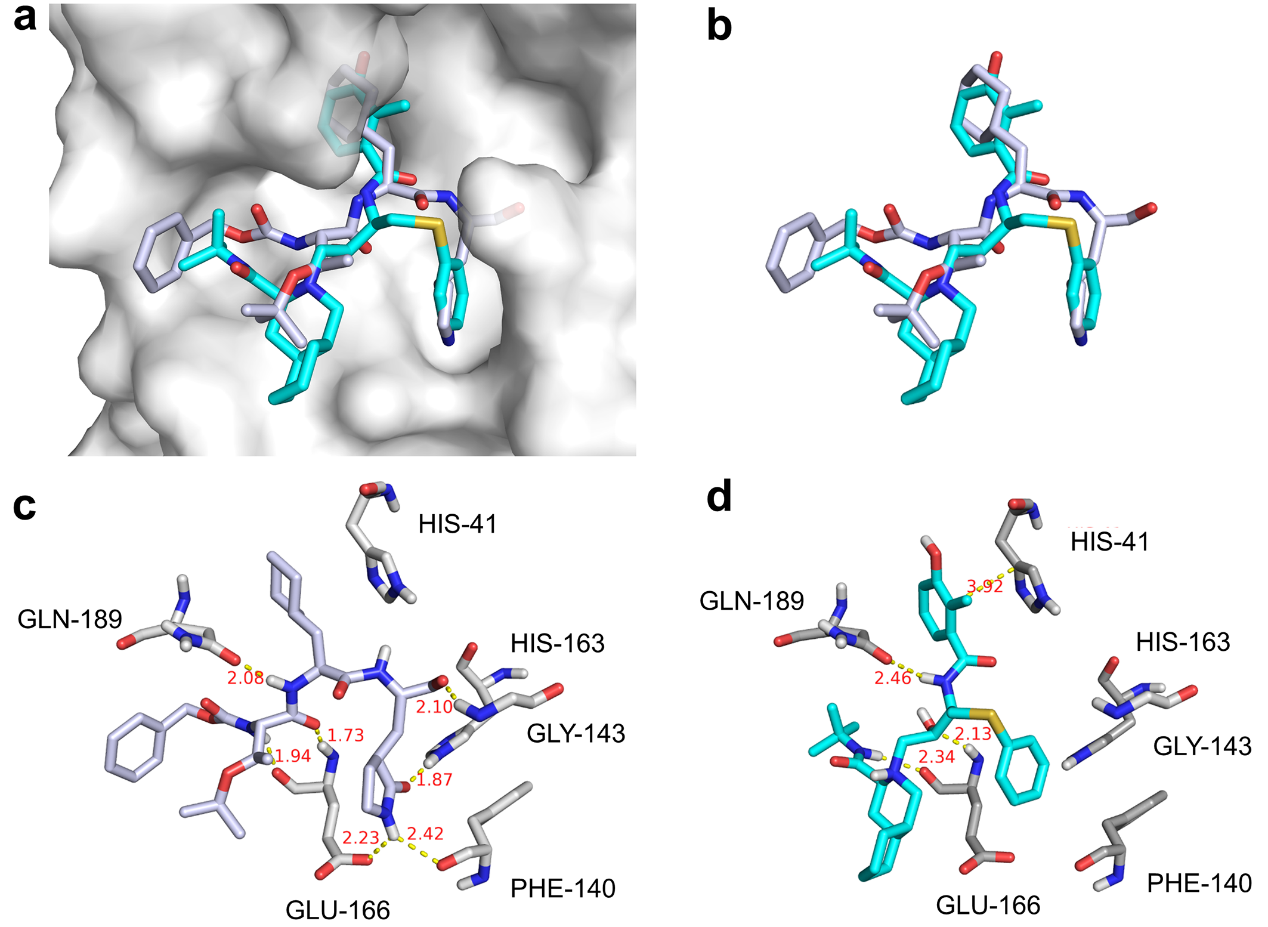


**Figure. S2.** The binding modes of nelfinavir against SARS-CoV-2 M^pro^. **a** Binding modes of the original ligand (TG-0205221, white) in 2GX4 and nelfinavir (cyan) in the SARS-CoV-2 M^pro^ protein pocket (white surface); **b** Superposition of TG-0205221 (white) and nelfinavir (cyan) in their binding conformations; **c** Interactions between TG-0205221 and associated residues in the crystal structure (2GX4) of SARS M^pro^; **d** Interactions between nelfinavir and associated residues in the homology model of SARS-CoV-2 M^pro^. The data in red is the interaction distance (Å).

**
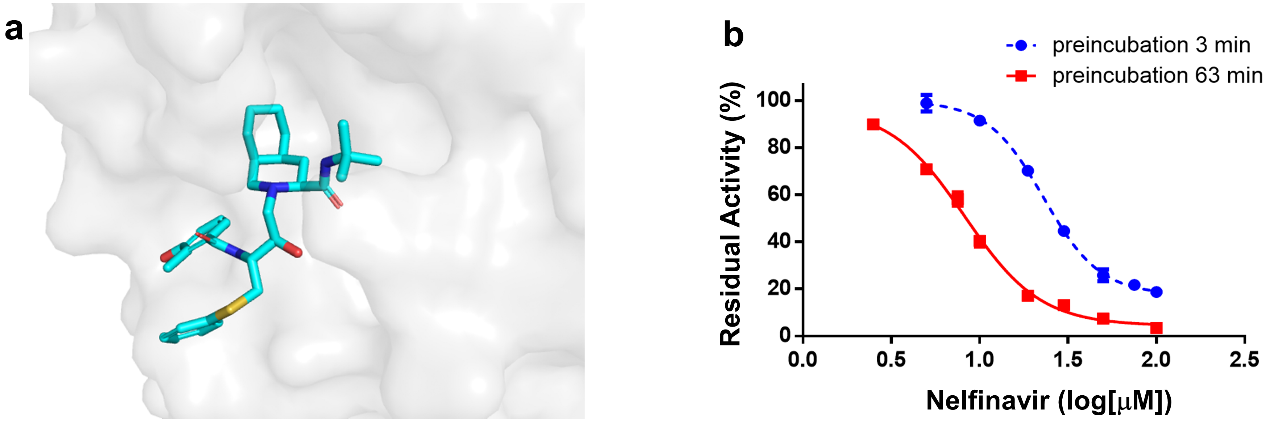
**

**Figure. S3.** *In silico* prediction and M^pro^ activities of nelfinavir. **a** Docking mode of nelfinavir (cyan) in the ligand binding pocket of SARS-CoV-2 M^pro^ (PDB ID: 7VH8). **b** Inhibitory activity of nelfinavir against SARS-CoV-2 M^pro^ by preincubation of 3 min and 63 min, respectively.


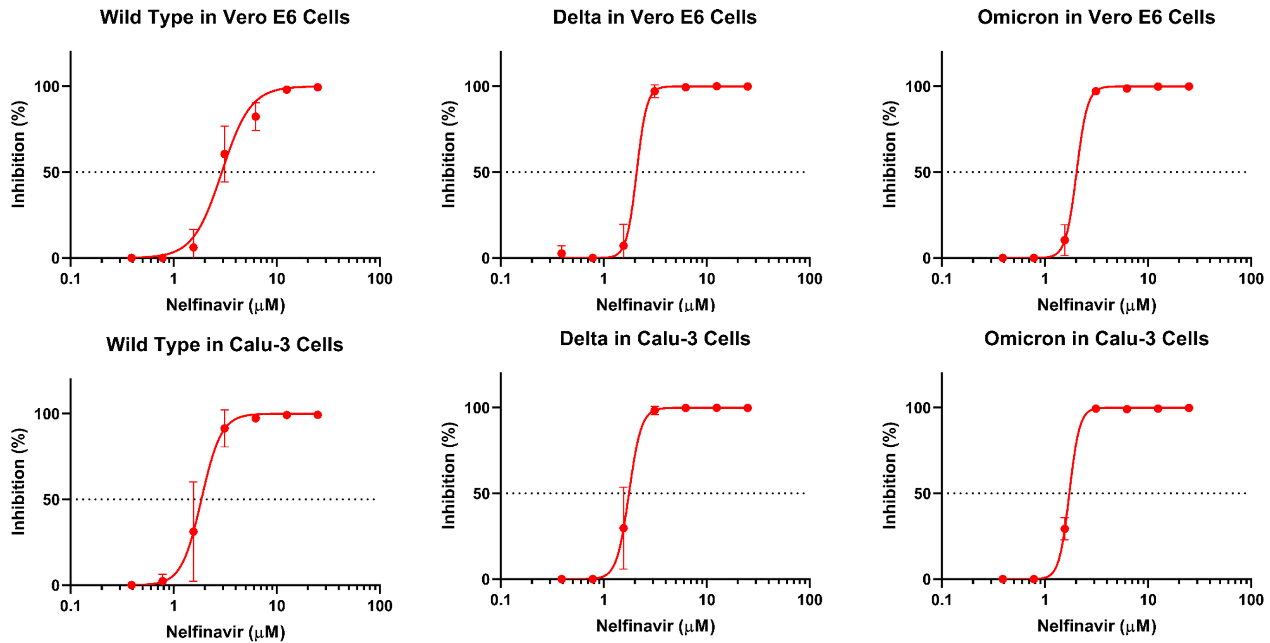


**Figure. S4.** *In vitro* inhibition of nelfinavir against wild type, Delta, Omicron SARS-CoV-2 in Vero E6 and Calu-3 cells.


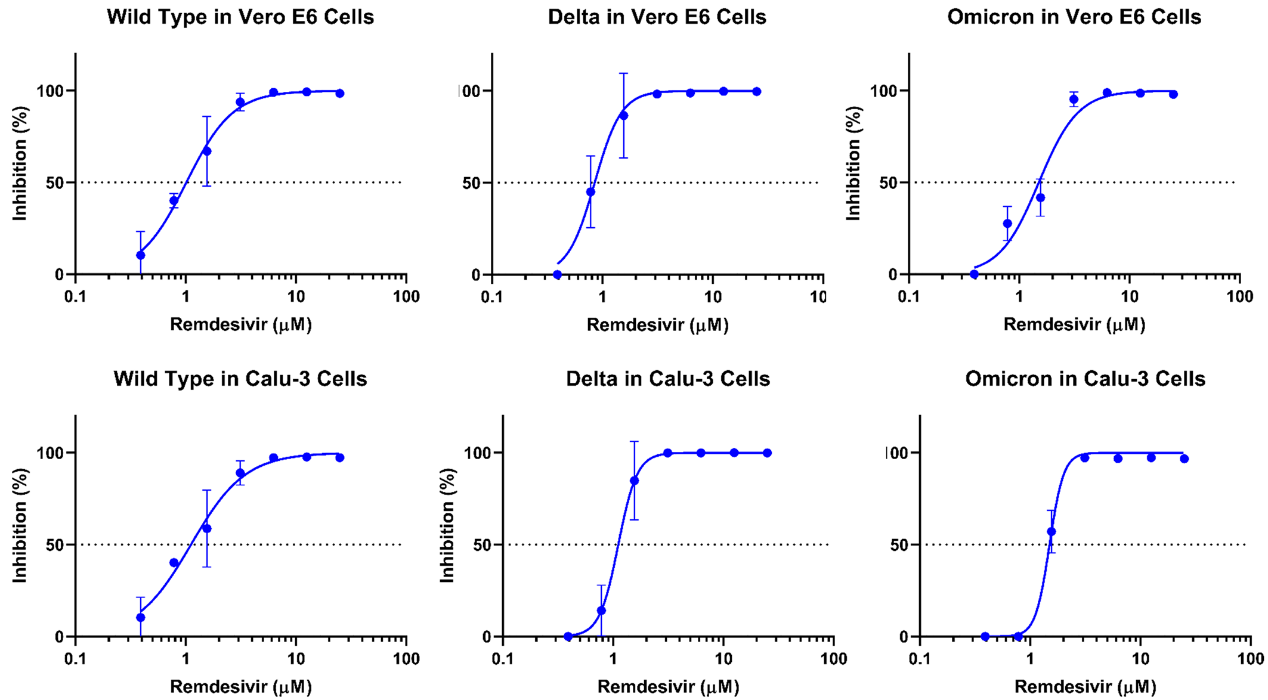


**Figure. S5.** *In vitro* inhibition of remdesivir against wild type, Delta, Omicron SARS-CoV-2 in Vero E6 and Calu-3 cells.


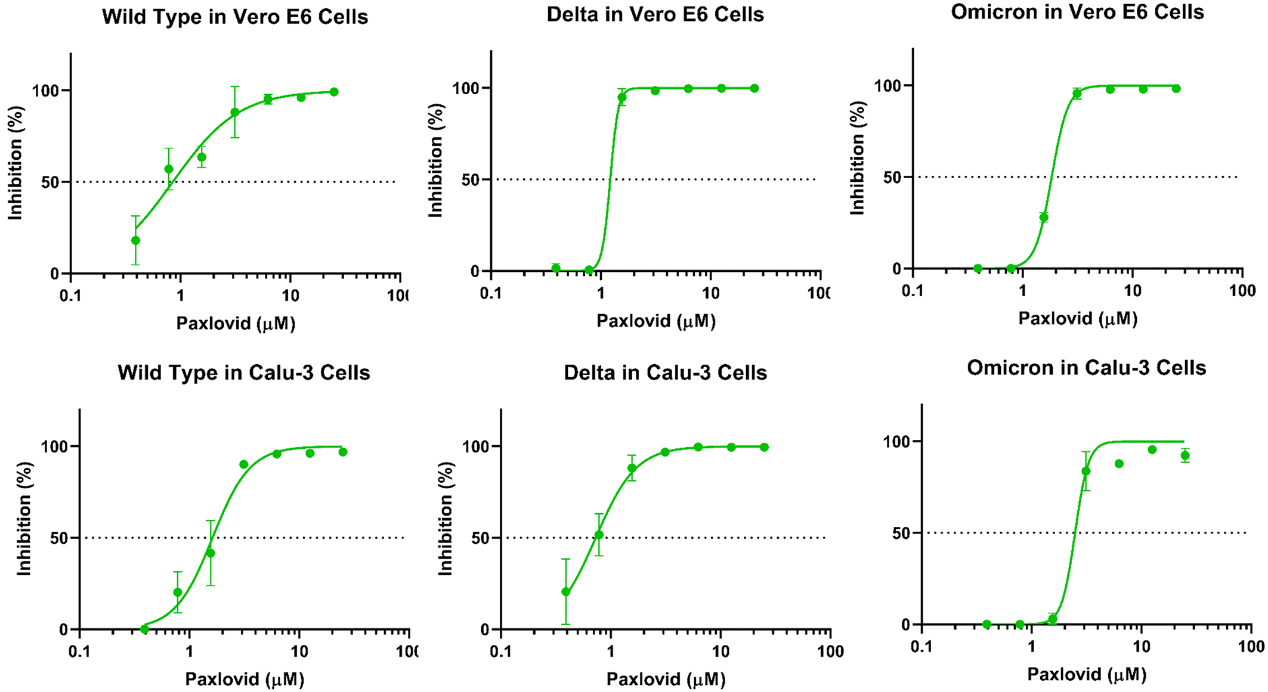


**Figure. S6.** *In vitro* inhibition of paxlovid against wild type, Delta, Omicron SARS-CoV-2 in Vero E6 and Calu-3 cells.


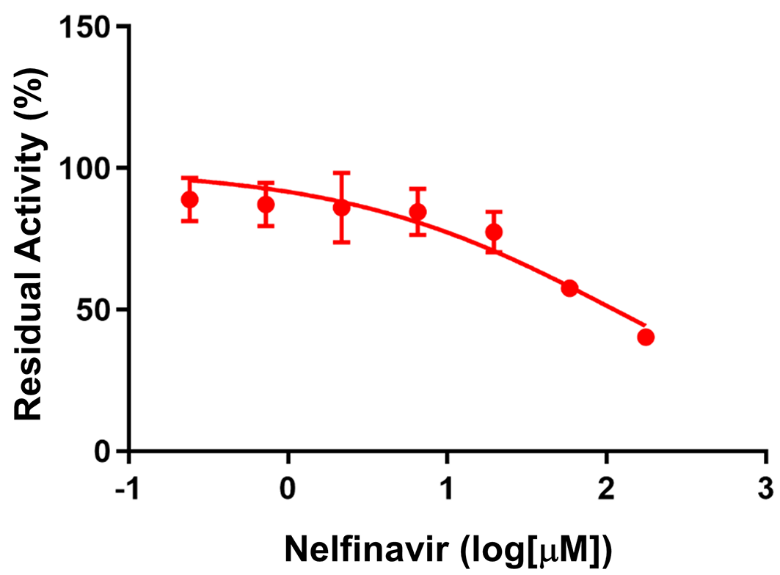


**Figure. S7.** The IC_50_ of nelfinavir against RdRp.

**Table S1.** Known M^pro^ binders as references to calculate the 3D similarity of binding modes.

| PDB ID | Ligand ID | Ligand Structure | Activity | Ref |
| --- | --- | --- | --- | --- |
| 1WOF | I12 |  | K_i_ = 10.7 μM | ^22^ |
| 2A5I | AZP |  | K_i_ = 18 μM | ^23^ |
| 2A5K | AZP |  | K_i_ = 18 μM | ^23^ |
| 2ALV | CY6 |  | IC_50_ = 70 μM | ^24^ |
| 2AMD | 9IN |  | K_i_ = 6.7 μM | ^22^ |
| 2GTB | AZP |  | k*inact*/K_i_ = 1900±400 M^−1^ s^−1^ | ^25^ |
| 2GX4 | NOL |  | Ki = 0.053 μM | ^21^ |
| 2GZ7 | D3F |  | IC_50_ = 0.3 μM | ^26^ |
| 2GZ8 | F3F |  | IC_50_ = 3 μM | ^26^ |
| 2OP9 | WR1 |  | K_i_ = 2.2 μM | ^27^ |
| 2QIQ | CYV |  | IC_50_ = 80 μM | ^28^ |
| 2V6N | XP1 |  | K_i_ = 1.38 μM | ^29^ |
| 2ZU4 | ZU3 |  | K_i_ = 0.038 μM | ^30^ |
| 2ZU5 | ZU5 |  | K_i_ = 0.099 μM | ^30^ |
| 3SN8 | S89 |  | K_i_ = 2.24 μM | ^31^ |
| 3SND | PRD_000772 |  | K_i_ = 8.27±1.52 μM | ^31^ |
| 3SZN | G75 |  | NA |  |
| 3TIT | G81 |  | NA |  |
| 3TIU | G82 |  | NA |  |
| 3TNS | G83 |  | NA |  |
| 3TNT | G85 |  | NA |  |
| 3V3M | 0EN |  | IC_50_ = 4.8 μM | ^32^ |
| 4F49 | K36 |  | IC_50_ = 0.82 μM | ^33^ |
| 4MDS | 23H |  | IC_50_ = 6.2 μM | ^34^ |
| 4TWW | 3A7 |  | IC_50_ = 63 μM | ^35^ |
| 4TWY | 3BL |  | IC_50_ = 108 μM | ^35^ |
| 4WY3 | 3X5 |  | IC_50_ = 240 μM | ^35^ |
| 4YLU | R30 |  | IC_50_ > 100 μM | ^36^ |
| 4YOG | 4F5 |  | IC_50_ = 1.8 μM | ^37^ |
| 4YOI | 4F4 |  | IC_50_ = 0.33 μM | ^37^ |
| 4YOJ | RFM |  | IC_50_ = 0.41 μM | ^37^ |
| 4ZRO | PRD_002174 |  | IC_50_ = 0.59 μM | ^38^ |
| 5C5N | SLH |  | NA |  |
| 5C5O | SDJ |  | NA |  |
| 5EU8 | PRD_002214 |  | NA |  |
| 5N5O | 8O5 |  | NA |  |
| 5N19 | D03 |  | NA |  |
| 5NH0 | 8X8 |  | NA |  |
| 5WKJ | B1S |  | EC_50_ = 0.9 μM* | ^39^ |
| 5WKK | AW4 |  | EC_50_ = 0.5 μM* | ^39^ |
| 5WKL | B3J |  | IC_50_ = 0.8 μM | ^39^ |
| 5WKM | N02 |  | IC_50_ = 6.1 μM | ^39^ |
| 6FV1 | E8E |  | NA |  |

* Racemic activity; NA: Not Available.

**Table S2.** 15 drugs selected from 2GTB model.

| DrugBank ID | Docking Score against 2GTB model (kcal/mol) | 3D Similarity Reference | 3D Similarity |
| --- | --- | --- | --- |
| DB00328 | -7.16 | 3V3M | 71.3% |
| DB13680 | -7.13 | 3SND | 70.8% |
| DB01165 | -7.31 | 3V3M | 70.2% |
| DB01198 | -7.81 | 5NH0 | 68.9% |
| DB08934 | -8.65 | 2GX4 | 68.6% |
| DB01165 | -7.31 | 5WKM | 68.3% |
| DB08860 | -8.06 | 2GTB | 68.1% |
| DB00402 | -7.75 | 5NH0 | 67.8% |
| DB08883 | -8.17 | 5NH0 | 67.1% |
| DB01288 | -7.96 | 5WKM | 67.1% |
| DB08860 | -8.06 | 2A5K | 66.9% |
| DB00972 | -7.71 | 5NH0 | 66.7% |
| DB00482 | -8.46 | 4YLU | 66.7% |
| DB00220 | -8.91 | 2GX4 | 66.4% |
| DB01058 | -7.90 | 3V3M | 65.7% |
| DB00328 | -7.16 | 4F49 | 65.6% |
| DB08934 | -8.65 | 3TNS | 65.5% |
| DB00904 | -7.45 | 5WKM | 65.4% |
| DB11951 | -8.27 | 4F49 | 65.3% |
| DB11951 | -8.27 | 5WKJ | 65.2% |

**Table S3.** 6 drugs selected from 10 new homology models.

| DrugBank ID | Name | Homology Template | Docking Score (kcal/mol) | 3D Similarity |
| --- | --- | --- | --- | --- |
| DB00220 | nelfinavir | 2GX4 | -9.18 | 70.2% |
| DB08860 | pitavastatin | 2GTB | -8.06 | 68.1% |
| DB08883 | perampanel | 5NH0 | -8.63 | 66.9% |
| DB01058 | praziquantel | 3V3M | -7.38 | 65.8% |
| DB01198 | zopiclone | 5NH0 | -8.46 | 63.5% |
| DB00402 | eszopiclone | 5NH0 | -8.46 | 63.5% |

**Table S4.** Components of the Binding Free Energy (kcal/mol) Calculated by MM/GBSA Approach^*^.

| Energy term | nelfinavir | pitavastatin | perampanel | praziquantel |
| --- | --- | --- | --- | --- |
| *E*_vdw_ | -58.36±0.32 | -42.77±0.30 | -44.33±0.25 | -34.92±0.32 |
| *E*_ele_ | -117.95±0.81 | 37.95±0.86 | 0.05±0.03 | -8.64±0.35 |
| *G*_gb_ | 134.49±0.83 | -22.40±1.63 | 14.76±0.29 | 21.53±0.32 |
| *G*_np_ | -6.69±0.03 | -5.60±0.03 | -5.43±0.03 | -4.34±0.03 |
| Δ*H* | -48.50±0.36 | -32.82±0.38 | -34.96±0.27 | -26.37±0.30 |
| TΔ*S* | -23.81±0.67 | -20.12±0.38 | -19.98±0.42 | -19.86±0.12 |
| Δ*G*_cal_ | -24.69±0.52 | -12.70±0.38 | -14.98±0.34 | -6.51±0.21 |

^*^: The statistical error was estimated based on 0.5-5 ns MD simulation trajectory. 500 snapshots evenly extracted from the 0.5-5 ns MD trajectory of complex were used for MM/GBSA calculations and 50 snapshots for the entropy term calculations.

**Table S5.** Components of the Binding Free Energy (kcal/mol) Calculated by SIE Approach^*^.

| Energy term | nelfinavir | pitavastatin | perampanel | praziquantel |
| --- | --- | --- | --- | --- |
| *E*_vdw_ | -58.36±0.32 | -42.77±0.30 | -44.33±0.25 | -34.92±0.32 |
| *E*_C_ | -52.44±0.36 | 16.87±0.82 | 0.02±0.01 | -3.84±0.16 |
| Δ*G*^R^_bind_ | 58.44±0.30 | -10.57±0.67 | 8.06±0.15 | 12.05±0.16 |
| *E*_cavity_ | -9.93±0.05 | -7.79±0.04 | -8.19±0.04 | -6.66±0.06 |
| Δ*G*_cal_ | -9.42±0.04 | -7.53±0.04 | -7.55±0.03 | -6.39±0.04 |

^*^: The binding free energy was calculated with the equation: Δ*G*_bind_ = 0.1048*(*E*_C_ + *E*_vdw_ + Δ*G*^R^_bind_ + *E*_cavity_) -2.89. *E*_cavity_=γΔ*MSA*. The statistical error was estimated based on 0.5-5 ns MD simulation trajectory. 500 snapshots evenly extracted from the 0.5-5 ns MD trajectory were used.

**Table S6.** Pharmacokinetic Parameters of Nelfinavir in 2 Healthy Rhesus Macaques after Nasogastric Administration at 200 mg/kg.

|  | Monkey 1 | Monkey 2 |
| --- | --- | --- |
| *C*max (nM) | 5325 | 1070 |
| *T*peak (h) | 6.00 | 4.00 |
| AUCon (nM*h) | 28408 | 10567 |
| AUCtot(nM*h) | 28450 | 10673 |
| *t*1/2 (h) | 4.46 | 6.92 |
| MRT (h) | 7.25 | 12.00 |

**Table S7.** Overview of Demographics and Baseline Characteristics (FAS).

|  | Nelfinavir (N=19) | Control (N=18) | Total | Statistics | *P* value | Method |
| --- | --- | --- | --- | --- | --- | --- |
| Age | | | | | | |
| Mean±SD | 39.2±9.11 | 37.7±15.24 | 38.5±12.32 | 0.358 | 0.723 | T'-test |
| Median | 38.0 | 34.0 | 38.0 |  |  |  |
| Q1～Q3* | 34.0～43.0 | 23.0～50.0 | 29.0～46.0 |  |  |  |
| Min～Max | 25～62 | 19～66 | 19～66 |  |  |  |
| Weight (kg) | | | | | | |
| N(NMiss) | 18(1) | 16(2) | 34(3) | 1.500 | 0.143 | T-test |
| Mean±SD | 67.42±11.49 | 62.47±6.85 | 65.09±9.78 |  |  |  |
| Median | 64.50 | 60.00 | 62.50 |  |  |  |
| Q1～Q3 | 59.00～75.00 | 57.50～66.00 | 58.00～70.00 |  |  |  |
| Min～Max | 54～93 | 55～80 | 54～93 |  |  |  |
| Sex | | | | | | |
| N | 19 | 18 | 37 | NA | 0.191 | Fisher |
| Men n(%) | 13(68.4) | 8(44.4) | 21(56.8) |  |  |  |
| Women n(%) | 6(31.6) | 10(55.6) | 16(43.2) |  |  |  |
| Ethnic group | | | | | | |
| N | 19 | 18 | 37 | NA | 0.604 | Fisher |
| Han n(%) | 18(94.7) | 16(88.9) | 34(91.9) |  |  |  |
| Other n(%) | 1(5.3) | 2(11.1) | 3(8.1) |  |  |  |
| Disease severity |  |  |  |  |  |  |
| N | 19 | 18 | 37 | NA | >0.999 | Fisher |
| mild n(%) | 14(73.7) | 14(77.8) | 28(75.7) |  |  |  |
| moderate n(%) | 5(26.3) | 4(22.2) | 9(24.3) |  |  |  |

*: Q1 and Q3 are the first and third quartiles of the data.

**Table S8.** Past medical history summarized according to SOC and PT (FAS) *

| SOC | Nelfinavir | |  | Control | | |  | Total | | | |  |
| --- | --- | --- | --- | --- | --- | --- | --- | --- | --- | --- | --- | --- |
| PT | Number (%) | Times | |  | Number (%) | Times | | |  | Number (%) | Times | |
| **N** | 19 |  | |  | 18 |  | | |  | 37 |  | |
| **Summary n(%)** | 10(52.6) | 20 | |  | 7(38.9) | 16 | | |  | 17(45.9) | 36 | |
| **Various examinations n(%)** | 8(42.1) | 12 | |  | 7(38.9) | 8 | | |  | 15(40.5) | 20 | |
| Lower blood calcium n(%) | 5(26.3) | 5 | |  | 4(22.2) | 4 | | |  | 9(24.3) | 9 | |
| Hypokalemia n(%) | 5(26.3) | 5 | |  | 4(22.2) | 4 | | |  | 9(24.3) | 9 | |
| Abnormal coagulation n(%) | 2(10.5) | 2 | |  | 0(0) | 0 | | |  | 2(5.4) | 2 | |
| **Vascular and lymphatic diseases**  **n(%)** | 2(10.5) | 2 | |  | 2(11.1) | 2 | | |  | 4(10.8) | 4 | |
| Hypertension n(%) | 2(10.5) | 2 | |  | 2(11.1) | 2 | | |  | 4(10.8) | 4 | |
| **Metabolic and nutritional diseases n(%)** | 1(5.3) | 1 | |  | 1(5.6) | 1 | | |  | 2(5.4) | 2 | |
| Hyperuricemia n(%) | 1(5.3) | 1 | |  | 0(0) | 0 | | |  | 1(2.7) | 1 | |
| Diabetes n(%) | 0(0) | 0 | |  | 1(5.6) | 1 | | |  | 1(2.7) | 1 | |
| **Hepatobiliary disease n(%)** | 1(5.3) | 1 | |  | 1(5.6) | 1 | | |  | 2(5.4) | 2 | |
| Abnormal liver function n(%) | 1(5.3) | 1 | |  | 1(5.6) | 1 | | |  | 2(5.4) | 2 | |
| **Respiratory, thoracic and mediastinal disorders n(%)** | 1(5.3) | 1 | |  | 1(5.6) | 2 | | |  | 2(5.4) | 3 | |
| Cough n(%) | 0(0) | 0 | |  | 1(5.6) | 1 | | |  | 1(2.7) | 1 | |
| Expectoration n(%) | 0(0) | 0 | |  | 1(5.6) | 1 | | |  | 1(2.7) | 1 | |
| Oropharyngeal pain n(%) | 1(5.3) | 1 | |  | 0(0) | 0 | | |  | 1(2.7) | 1 | |
| **Mental illness n(%)** | 1(5.3) | 1 | |  | 1(5.6) | 1 | | |  | 2(5.4) | 2 | |
| Insomnia n(%) | 1(5.3) | 1 | |  | 1(5.6) | 1 | | |  | 2(5.4) | 2 | |
| **Infectious Diseases n(%)** | 1(5.3) | 1 | |  | 0(0) | 0 | | |  | 1(2.7) | 1 | |
| Hepatitis virus carriers n(%) | 1(5.3) | 1 | |  | 0(0) | 0 | | |  | 1(2.7) | 1 | |
| **Gastrointestinal diseases n(%)** | 1(5.3) | 1 | |  | 0(0) | 0 | | |  | 1(2.7) | 1 | |
| Diarrhea n(%) | 1(5.3) | 1 | |  | 0(0) | 0 | | |  | 1(2.7) | 1 | |
| **Heart disease n(%)** | 0(0) | 0 | |  | 1(5.6) | 1 | | |  | 1(2.7) | 1 | |
| Coronary arteriosclerosis n(%) | 0(0) | 0 | |  | 1(5.6) | 1 | | |  | 1(2.7) | 1 | |

*: SOC (System Organ Class), PT (Preferred Term).

**Table S9.** The time (d) to convert to negative throat swab of SARS-CoV-2 by log-rank analysis.

|  | Nelfinavir | Control | Statistics | *P* value |
| --- | --- | --- | --- | --- |
| N | 19 | 18 | 3.688 | 0.055 |
| Events(%) | 19 | 18 |  |  |
| 25%(95%CI) | 5.0(1.0~9.0) | 6.0(2.0~12.0) |  |  |
| 50%(95%CI) | 9.0(5.0~11.0) | 14.5(6.0~25.0) |  |  |
| 75%(95%CI) | 12.0(9.0~22.0) | 27.0(15.0~34.0) |  |  |

**Table S10.** The time (d) of TTCR by log-rank analysis.

|  | Nelfinavir | Control | Statistics | *P* value |
| --- | --- | --- | --- | --- |
| N(NMiss) ^*^ | 5(14) | 6(12) | 0.016 | 0.899 |
| Censored(%) | 2(40.0) | 2(33.3) |  |  |
| Events | 3 | 4 |  |  |
| 25%(95%CI) | 4.0(1.0~ NE) | 6.0(2.0~12.0) |  |  |
| 50%(95%CI) | 7.0(1.0~ NE) | 11.0(2.0~ NE) |  |  |
| 75%(95%CI) | NE(1.0~ NE) | NE(6.0~ NE) |  |  |

^*^: Only patients with abnormal body temperature, respiratory rate, or oxygen saturation at baseline were analyzed.

**Table S11.** Temperatures in the control group before treatment (days -1 ~ days-5), days 1~14, and day 14+3.

| Patients No. | D-1~-5 | D1 | D2 | D3 | D4 | D5 | D6 | D7 | D8 | D9 | D10 | D11 | D12 | D13 | D14 | D14+3 |
| --- | --- | --- | --- | --- | --- | --- | --- | --- | --- | --- | --- | --- | --- | --- | --- | --- |
| 01 | 36.7 | 36.7 | 36.7 | 36.5 | 36.5 | 36.4 | 36.8 | 36.6 | 36.8 | 36.7 | 36.6 |  | 36.6 |  | 37.0 |  |
| 02 | 36.1 | 36.5 | 36.5 | 36.4 | 37.8 | 37.6 | 37.2 | 37.2 |  |  |  |  |  |  |  |  |
| 03 |  | 37.2 | 37.5 | 37.4 | 37.3 | 37.1 | 37.4 | 37.4 | 36.4 | 37.5 | 37.2 | 37.4 | 36.8 | 36.8 | 37.4 | 36.5 |
| 04 | 36.6 | 36.3 | 36.2 | 36.3 | 36.1 | 36.5 |  |  |  |  |  |  |  |  |  |  |
| 05 | 36.5 | 36.3 | 36.5 | 36.5 | 36.5 | 36.6 | 36.4 | 36.3 | 36.1 | 36.3 |  | 36.3 | 36.3 | 36.8 | 36.9 |  |
| 06 | 36.6 | 36.4 | 36.6 | 36.6 | 36.5 | 36.5 | 36.3 | 36.6 | 36.3 | 36.2 | 36.3 | 36.3 | 36.9 | 36.9 | 36.9 |  |
| 07 | 36.3 | 37.5 | 37.8 | 37.7 | 37.7 | 37.1 | 36.9 | 36.6 | 36.6 | 36.8 | 37 | 36.9 | 37 | 36.7 | 36.9 |  |
| 08 | 37 | 37 | 37.8 | 37.7 | 37.7 | 37.1 | 36.0 | 36.5 | 36.2 | 36.5 | 36.6 | 36.9 | 36.1 | 36.1 | 36.4 | 36.8 |
| 09 | 36.4 | 36.4 | 36.4 | 36.4 | 37.8 | 37.2 | 36.1 | 36 | 36.6 | 36.5 | 36 | 36.4 | 36.6 | 36.6 | 36.9 |  |
| 10 | 37.2 | 36.3 | 36.4 | 36.3 | 37.2 | 36.7 | 36.5 | 36.6 | 37.2 | 37.1 | 37 | 36.4 | 36.9 | 36.7 | 36.7 |  |
| 11 | 37 | 37.2 | 36.7 | 36.6 | 36.5 | 36.4 | 36.9 | 36.7 | 36.4 | 36.9 | 36.7 | 36.9 | 36.7 | 36.3 | 36.9 |  |
| 12 | 37.6 | 38.6 | 39.2 | 38.6 | 38.6 | 37.9 | 38 | 37.4 | 37.7 | 37.2 | 36.6 | 36.4 | 36.6 | 36.6 | 36.5 |  |
| 13 | 37 | 36.6 | 36.8 | 36.7 | 36.6 | 37.1 | 37.3 | 37.1 | 37.2 | 37.3 | 37 | 36.9 | 36.8 | 36.5 | 36.3 | 37 |
| 14 | 37.1 | 37.2 | 36.6 | 38 | 37.4 | 36.3 | 37.5 | 37.1 | 37.3 | 37.1 | 36.4 | 36.8 | 37.2 | 36.7 | 37 |  |
| 15 | 36.6 | 36.5 | 36.4 | 36.3 | 36.1 | 36.1 |  |  |  |  |  |  |  |  |  |  |
| 16 | 37.8 | 36.2 | 38.2 | 37.6 | 37.8 | 37.6 | 37.8 | 37.4 | 37.1 | 37.3 | 36.8 | 37.1 | 36.7 | 36.6 | 36.9 |  |
| 17 | 37.6 | 37.5 | 37.5 | 37.6 | 37.4 | 37.5 | 37.4 | 37.6 | 37.3 | 37.3 | 37.3 | 37.5 | 37.5 | 37.5 | 37.3 |  |
| 18 | 38.4 | 38.5 | 39 | 37.4 | 37.1 | 37.1 | 36.3 | 36.5 | 36.7 | 37 | 36.5 | 36.8 | 36.7 | 36.7 | 36.6 |  |

**Table S12.** Temperatures in the nelfinavir group before treatment (days -1 ~ days-5), days 1~14, and day 14+3.

| Patients No. | D-1~-5 | D1 | D2 | D3 | D4 | D5 | D6 | D7 | D8 | D9 | D10 | D11 | D12 | D13 | D14 | D14+3 |
| --- | --- | --- | --- | --- | --- | --- | --- | --- | --- | --- | --- | --- | --- | --- | --- | --- |
| 01 | 36.6 | 36.7 | 36.3 | 36.5 | 36.5 | 36.4 | 36.5 | 36.5 | 36.4 | 36.6 | 36.5 | 36.5 | 36.5 | 36.4 | 37.8 |  |
| 02 | 38.2 | 39.2 | 37.3 | 37.4 | 36.8 | 36.7 | 36.6 |  | 37 |  |  |  |  |  |  |  |
| 03 | 36.1 | 36.7 | 36.3 | 36.1 | 36.2 | 36.4 | 36.2 | 36.3 | 36.3 | 36.6 | 36.6 | 36.5 |  |  |  |  |
| 04 | 36.1 | 36.3 | 36.1 | 36.3 | 36.6 | 36.5 | 36.1 | 36.5 | 36.2 | 36.3 | 36.3 | 36.2 | 36.3 | 36.3 | 36.4 |  |
| 05 | 36.3 | 36.2 | 37 | 36.3 | 36.6 | 36.9 | 36.3 | 36.6 | 36.3 | 36.3 |  |  |  |  |  |  |
| 06 | 36.1 | 36.3 | 36.3 | 36.1 | 36.3 | 36.4 | 36.4 | 36.3 | 36.7 | 36.3 | 36.6 | 36.4 | 36.8 | 36.4 | 36.8 |  |
| 07 | 36.5 | 36.3 | 36.3 | 36.3 | 36.3 | 36.3 | 37 | 36.8 | 36.9 | 37 | 36.6 | 36.8 | 36.9 | 36.6 | 36.3 |  |
| 08 | 36.3 | 36.3 | 36.2 | 36.3 | 36.3 | 36.3 | 36.3 | 37.1 | 36.8 | 37.3 | 37 | 37 | 36.9 | 36.7 | 36.9 |  |
| 09 | 36.4 | 36.3 | 36.4 | 36.2 | 36.9 | 36.7 | 36.8 | 36.6 |  |  |  |  |  |  |  |  |
| 10 | 36 | 36.5 | 36.6 | 36.7 | 36.4 | 36.5 | 36.5 | 36.2 | 36.2 | 36.5 | 36.8 | 36.8 | 36.8 | 36.7 | 36.4 |  |
| 11 | 36.7 | 37.1 | 36.3 | 37.2 | 37.2 | 37 | 36.6 | 37 | 37 | 36.7 | 36.4 | 36.5 | 36.1 | 36.4 | 36.5 | 37.3 |
| 12 | 37 | 37.3 | 37 | 36.7 | 37.1 | 36.9 | 37.1 | 37 | 37 | 36.6 | 37.1 | 36.5 | 36.1 | 37.1 | 36.1 |  |
| 13 | 36.5 | 36.6 | 36.9 | 36.3 | 36.6 | 36.3 | 36.6 | 36.8 | 36.9 | 36.7 | 37.1 | 36.9 | 36.7 | 37 | 36.5 | 36.6 |
| 14 | 37 | 36.6 | 36.7 | 36.6 | 36.9 | 36.7 | 37 | 36.4 | 36.7 | 36.9 | 36.6 | 36.6 | 36.3 | 36.8 | 36.7 |  |
| 15 | 36.6 |  |  |  |  |  |  |  |  |  |  |  |  |  |  |  |
| 16 | 37.2 | 37.5 | 37.2 | 36.3 | 36.4 | 36.5 | 36.5 | 36.6 | 36.6 | 36.5 | 36.8 | 36.3 | 36.5 | 36.6 | 36.6 |  |
| 17 | 36.4 | 36.8 | 36.6 | 36.6 | 37 | 36.4 | 36.5 | 37.3 | 36.6 | 36.3 |  |  |  |  |  |  |
| 18 | 37.8 | 36.9 | 37 | 37.1 | 36.9 | 36.7 | 36.6 | 36.7 | 37 | 37.1 | 36.9 | 36.9 | 37.3 | 37 | 36.7 |  |
| 19 | 37.2 | 36.9 | 36.1 | 36.7 | 36.4 | 36.5 |  |  |  |  |  |  |  |  |  |  |

**Table S13.** The lung-to-plasma nelfinavir concentration ratios in mouse after administered orally (n=3).

| Dose/Time | 0.5h | 1h | 3h | 6h | 9h |
| --- | --- | --- | --- | --- | --- |
| 250 mg/kg | 2.08±0.50 | 2.35±0.34 | 2.24±0.59 | 1.81±0.19 | 2.33±0.28 |
| 750mg/kg | 2.07±0.42 | 3.11±0.82 | 2.83±0.33 | 3.22±0.63 | 2.61±0.08 |

**References**

1 Schrodinger, LLC. *The PyMOL Molecular Graphics System*, Version 2.4 (2019).

2 Waterhouse, A. *et al.* SWISS-MODEL: homology modelling of protein structures and complexes. *Nucleic Acids Res.* **46**, W296-W303 (2018).

3 Wishart, D. S. *et al.* DrugBank 5.0: a major update to the DrugBank database for 2018. *Nucleic Acids Res.* **46**, D1074-D1082 (2018).

4 Morris, G. M. *et al.* AutoDock4 and AutoDockTools4: Automated docking with selective receptor flexibility. *J. Comput. Chem.* **30**, 2785-2791 (2009).

5 Koes, D. R., Baumgartner, M. P. & Camacho, C. J. Lessons Learned in Empirical Scoring with smina from the CSAR 2011 Benchmarking Exercise. *J. Chem. Inf. Model.* **53**, 1893-1904 (2013).

6 Trott, O. & Olson, A. J. AutoDock Vina: Improving the speed and accuracy of docking with a new scoring function, efficient optimization, and multithreading. *J. Comput. Chem.* **31**, 455-461 (2010).

7 Dolinsky, T. J., Nielsen, J. E., McCammon, J. A. & Baker, N. A. PDB2PQR: an automated pipeline for the setup of Poisson-Boltzmann electrostatics calculations. *Nucleic Acids Res.* **32**, W665-667 (2004).

8 Vaz de Lima, L. A. & Nascimento, A. S. MolShaCS: a free and open source tool for ligand similarity identification based on Gaussian descriptors. *Eur. J. Med. Chem.* **59**, 296-303 (2013).

9 Wang, J., Wolf, R. M., Caldwell, J. W., Kollman, P. A. & Case, D. A. Development and testing of a general amber force field. *J. Comput. Chem.* **25**, 1157-1174 (2004).

10 Duan, Y. *et al.* A point-charge force field for molecular mechanics simulations of proteins based on condensed-phase quantum mechanical calculations. *J. Comput. Chem.* **24**, 1999-2012 (2003).

11 Kollman, P. A. *et al.* Calculating structures and free energies of complex molecules: combining molecular mechanics and continuum models. *Acc. Chem. Res.* **33**, 889-897 (2000).

12 Srinivasan, J., Cheatham, T. E., Cieplak, P., Kollman, P. A. & Case, D. A. Continuum Solvent Studies of the Stability of DNA, RNA, and Phosphoramidate−DNA Helices. *J. Am. Chem. Soc.* **120**, 9401-9409 (1998).

13 Naïm, M. *et al.* Solvated Interaction Energy (SIE) for Scoring Protein−Ligand Binding Affinities. 1. Exploring the Parameter Space. *J. Chem. Inf. Model.* **47**, 122-133 (2007).

14 Onufriev, A., Bashford, D. & Case, D. A. Exploring protein native states and large-scale conformational changes with a modified generalized born model. *Proteins* **55**, 383-394 (2004).

15 Xue, X. *et al.* Production of Authentic SARS-CoV Mpro with Enhanced Activity: Application as a Novel Tag-cleavage Endopeptidase for Protein Overproduction. *J. Mol. Biol.* **366**, 965-975 (2007).

16 Studier, F. W. Protein production by auto-induction in high-density shaking cultures. *Protein Expression Purif.* **41**, 207-234 (2005).

17 Chen, L. *et al.* Discovering Severe Acute Respiratory Syndrome Coronavirus 3CL Protease Inhibitors: Virtual Screening, Surface Plasmon Resonance, and Fluorescence Resonance Energy Transfer Assays. *J. Biomol. Screen.* **11**, 915-921 (2006).

18 Xiong, Y. *et al.* Flavonoids in Ampelopsis grossedentata as covalent inhibitors of SARS-CoV-2 3CLpro: Inhibition potentials, covalent binding sites and inhibitory mechanisms. *Int. J. Biol. Macromol.* **187**, 976-987 (2021).

19 Yao, H. *et al.* Molecular Architecture of the SARS-CoV-2 Virus. *Cell* **183**, 730-738.e713 (2020).

20 Chen, Z. *et al.* D3Pockets: A Method and Web Server for Systematic Analysis of Protein Pocket Dynamics. *J. Chem. Inf. Model.* **59**, 3353-3358 (2019).

21 Yang, S. *et al.* Synthesis, crystal structure, structure-activity relationships, and antiviral activity of a potent SARS coronavirus 3CL protease inhibitor. *J. Med. Chem.* **49**, 4971-4980 (2006).

22 Yang, H. *et al.* Design of Wide-Spectrum Inhibitors Targeting Coronavirus Main Proteases. *PLoS Biol.* **3**, e324 (2005).

23 Lee, T.-W. *et al.* Crystal Structures of the Main Peptidase from the SARS Coronavirus Inhibited by a Substrate-like Aza-peptide Epoxide. *J. Mol. Biol.* **353**, 1137-1151 (2005).

24 Ghosh, A. K. *et al.* Design and Synthesis of Peptidomimetic Severe Acute Respiratory Syndrome Chymotrypsin-like Protease Inhibitors. *J. Med. Chem.* **48**, 6767-6771 (2005).

25 Lee, T.-W. *et al.* Crystal Structures Reveal an Induced-fit Binding of a Substrate-like Aza-peptide Epoxide to SARS Coronavirus Main Peptidase. *J. Mol. Biol.* **366**, 916-932 (2007).

26 Lu, I. L. *et al.* Structure-Based Drug Design and Structural Biology Study of Novel Nonpeptide Inhibitors of Severe Acute Respiratory Syndrome Coronavirus Main Protease. *J. Med. Chem.* **49**, 5154-5161 (2006).

27 Goetz, D. H. *et al.* Substrate Specificity Profiling and Identification of a New Class of Inhibitor for the Major Protease of the SARS Coronavirus. *Biochemistry* **46**, 8744-8752 (2007).

28 Ghosh, A. K. *et al.* Structure-based design, synthesis, and biological evaluation of peptidomimetic SARS-CoV 3CLpro inhibitors. *Bioorg. Med. Chem. Lett.* **17**, 5876-5880 (2007).

29 Verschueren, K. H. G. *et al.* A Structural View of the Inactivation of the SARS Coronavirus Main Proteinase by Benzotriazole Esters. *Chem. Biol.* **15**, 597-606 (2008).

30 Lee, C.-C. *et al.* Structural Basis of Inhibition Specificities of 3C and 3C-like Proteases by Zinc-coordinating and Peptidomimetic Compounds. *J. Biol. Chem.* **284**, 7646-7655 (2009).

31 Zhu, L. *et al.* Peptide aldehyde inhibitors challenge the substrate specificity of the SARS-coronavirus main protease. *Antiviral Res.* **92**, 204-212 (2011).

32 Jacobs, J. *et al.* Discovery, Synthesis, And Structure-Based Optimization of a Series of *N*-(*tert*-Butyl)-2-(*N*-arylamido)-2-(pyridin-3-yl) Acetamides (ML188) as Potent Noncovalent Small Molecule Inhibitors of the Severe Acute Respiratory Syndrome Coronavirus (SARS-CoV) 3CL Protease. *J. Med. Chem.* **56**, 534-546 (2013).

33 Kim, Y. *et al.* Broad-Spectrum Antivirals against 3C or 3C-Like Proteases of Picornaviruses, Noroviruses, and Coronaviruses. *J. Virol.* **86**, 11754-11762 (2012).

34 Turlington, M. *et al.* Discovery of *N*-(benzo[1,2,3]triazol-1-yl)-*N*-(benzyl)acetamido)phenyl) carboxamides as severe acute respiratory syndrome coronavirus (SARS-CoV) 3CLpro inhibitors: Identification of ML300 and noncovalent nanomolar inhibitors with an induced-fit binding. *Bioorg. Med. Chem. Lett.* **23**, 6172-6177 (2013).

35 Shimamoto, Y. *et al.* Fused-ring structure of decahydroisoquinolin as a novel scaffold for SARS 3CL protease inhibitors. *Biorg. Med. Chem.* **23**, 876-890 (2015).

36 Tomar, S. *et al.* Ligand-induced Dimerization of Middle East Respiratory Syndrome (MERS) Coronavirus nsp5 Protease (3CL^pro^): IMPLICATIONS FOR nsp5 REGULATION AND THE DEVELOPMENT OF ANTIVIRALS. *J. Biol. Chem.* **290**, 19403-19422 (2015).

37 St. John, S. E., Tomar, S., Stauffer, S. R. & Mesecar, A. D. Targeting zoonotic viruses: Structure-based inhibition of the 3C-like protease from bat coronavirus HKU4—The likely reservoir host to the human coronavirus that causes Middle East Respiratory Syndrome (MERS). *Biorg. Med. Chem.* **23**, 6036-6048 (2015).

38 St. John, S. E. *et al.* X-ray structure and inhibition of the feline infectious peritonitis virus 3C-like protease: Structural implications for drug design. *Bioorg. Med. Chem. Lett.* **25**, 5072-5077 (2015).

39 Galasiti Kankanamalage, A. C. *et al.* Structure-guided design of potent and permeable inhibitors of MERS coronavirus 3CL protease that utilize a piperidine moiety as a novel design element. *Eur. J. Med. Chem.* **150**, 334-346 (2018).
